# Age-related differences in axon pruning and myelination may alter neural signaling in autism spectrum disorder

**DOI:** 10.1186/s13229-025-00684-y

**Published:** 2025-10-23

**Authors:** Kari L. Hanson, Thomas Avino, Sandra L. Taylor, Karl D. Murray, Cynthia M. Schumann

**Affiliations:** 1 Department of Psychiatry and Behavioral Sciences, School of Medicine, University of California, Davis, Sacramento, CA USA; 2 MIND Institute, School of Medicine, University of California, Davis, 2805 50th St, Sacramento, CA 95817 USA; 3 Department of Public Health Sciences, School of Medicine, University of California, Davis, CA USA; 4 Department of Physiology and Membrane Biology, School of Medicine, University of California, Davis, CA USA; 5 Department of Cell Biology and Human Anatomy, School of Medicine, University of California, Davis, CA USA

**Keywords:** Autism, Myelin, Axon density, White matter, Brain development

## Abstract

**Background:**

Neuronal connectivity is refined throughout development by the proliferation and pruning of axons in cerebral white matter, and progressive axon myelination that enables rapid communication across brain regions. Differences in connectivity have been observed in autism spectrum disorder (ASD), including changes in white matter volume and connectivity. In the prefrontal cortex, this includes imbalances between short- and long-ranging axons, consistent with a pattern of local hyperconnectivity, and long-range hypoconnectivity. Alterations in temporal lobe white matter development—critical for social behavior—may contribute to atypical neural connectivity.

**Methods:**

We used electron microscopy to analyze 54 samples of temporal lobe white matter from 27 age-matched postmortem brains from males with ASD and neurotypical (NT) controls, ages 2–44 years. Defined regions of superficial (SWM) and deep (DWM) white matter were sampled from superior temporal (STG) and fusiform (FG) gyri. Axon density and myelin thickness were quantified, with axon size classified by inner diameter, to evaluate age-related differences between ASD and neurotypical brains.

**Results:**

In neurotypical control brains, total axon density significantly decreases with age in both STG and FG SWM. Although ASD cases show a similar trend, the density of small axons in STG is significantly higher than in controls. However, FG SWM in ASD shows no significant change in small-diameter axon density with age in this region. In neurotypical brains, myelin thickness of large-diameter axons increases significantly with age in STG and FG SWM. In contrast, large-diameter axons in ASD display significantly thinner myelin sheaths than controls across both STG and FG regions.

**Conclusions:**

The temporal lobe exhibits atypical patterns of white matter development in ASD. In neurotypical individuals, decreased axon density in SWM with age reflects effective neural pruning and refinement of local and short-range connectivity. In contrast, individuals with ASD maintain a high density of small-diameter axons in STG SWM, suggesting reduced pruning that results in local overconnectivity. Moreover, myelin thickness in SWM does not increase with age in ASD, implying reduced efficacy of neurotransmission. These alterations in white matter ultrastructure may contribute to the atypical connectivity and neural communication observed in ASD across the lifespan.

**Supplementary Information:**

The online version contains supplementary material available at 10.1186/s13229-025-00684-y.

## Background

The human brain is shaped by complex developmental processes, evolving from basic networks of neural activity into a sophisticated orchestration of interconnected systems via postnatal changes in cell number, axon density, and myelination [1–7]. Developing cortical white matter is comprised of axons that support neural communication across local and distal cortical brain regions. White matter volumes change dynamically across the lifespan, with rapid growth in early to mid-gestation that continues into adolescence, followed by a gradual decline later in life [8, 9]. These volumetric changes reflect structural growth and reorganization driven by synaptogenesis, axonogenesis, and activity-dependent pruning—processes that refine and strengthen neural connectivity. Myelination by oligodendrocytes enhances the speed and efficiency of neural transmission that continues through the first few decades of life [10–12], with modest decline in healthy aging adults [6].

Within cortical white matter, axons localized to superficial white matter (SWM), sub-adjacent to cortical gray matter, primarily integrate local cortical circuits, while those in deep white matter (DWM) link to more distal cortical regions and subcortical structures. This spatial distinction between local versus long-range connectivity is essential for supporting complex sensory-motor and cognitive functions. Histological studies reveal dramatic reorganization of white matter ultrastructure in typically developing adults [1, 13, 14], including pruning of small-diameter axons connecting nearby regions, and progressive thickening of myelin sheaths around large-diameter axons connecting to distal and subcortical regions [15, 16].

Autism Spectrum Disorder (ASD) is a neurodevelopmental condition characterized by challenges in social interaction and communication along with restricted and repetitive behaviors that emerge in early childhood and persist throughout life [17]. While the cellular mechanisms thought to underlie ASD are diverse and heterogeneous, extensive studies show deviations from the neurotypical maturation of cellular connectivity [18–26]. Neuroimaging studies frequently report reduced functional connectivity in individuals with ASD [27–31], notably involving trade-offs in local and long-range connectivity [28, 32].

Diffusion Tensor Imaging (DTI) studies consistently report altered axonal tract organization in individuals with ASD [33–37]. Reductions in fractional anisotropy (FA), a DTI measure of directional water diffusion, suggest disrupted white matter fiber integrity [29, 38–48], notably in temporal lobe and the corpus callosum [33, 47, 49, 50]. Myelin water fraction (MWF), an MRI-based index of myelin integrity, is also reduced in young adults with ASD [51], indicating that changes in axon structure and myelination emerge in late childhood or early adolescence and persist into adulthood.

While DTI measures provide important clues, they lack specificity and can be influenced by multiple factors including axon diameter, density, and myelination [52, 53]. Direct ultrastructural examination of postmortem tissue can more precisely reveal the cellular basis of white matter alterations in ASD. Studies of frontal cortical white matter suggest a departure from typical myelination patterns and alterations in axonal organization of short- and long-ranging axons [20, 21], suggesting that ASD is associated with developmental changes in neural connectivity, characterized by local hyperconnectivity of short-range axons and decreased connectivity of long-range axons between regions [35, 54, 55].

The temporal lobe is consistently implicated in pathophysiology of ASD and associated deficits in language and social communication [56–59]. DTI studies have shown white matter impairments in social and language pathways in the temporal lobe in ASD [37], and changes to white matter ultrastructure correlate with the degree of social impairment in the disorder [34]. To examine the underlying cellular basis of white matter alterations in temporal lobe, we conducted ultrastructural analyses using electron microscopy (EM) in 27 postmortem brains from males with and without ASD, aged 2 to 44 years. This high-resolution approach enabled quantification of axon density and myelination across development, providing a cellular framework for interpreting neuroimaging findings [60, 61] and advancing understanding of the developmental trajectory of neural connectivity in ASD.

## Materials and methods

### Case information

Supplementary Table S1 lists subject information for the human brain tissue utilized in this study. Postmortem samples of the temporal lobe were obtained from NIH Neurobiobank and the Autism Tissue Program, now Autism Brain Net. Brain tissue was procured from donors with informed consent from next of kin [62] in accordance with standard procedures approved by the brain banks’ scientific review committees and Institutional Review Boards (IRBs). A total of 54 samples from 27 all-male cases were analyzed, including 14 individuals with a diagnosis of ASD (ages 5–44, average age 21.86 years, SD = 12.68) and 13 neurotypical individuals (ages 2–44, average age 20.31, SD = 11.29). Student’s 2-tailed t tests revealed no significant differences in postmortem interval (PMI) between ASD (21.85 h, SD = 11.60) and NT (22.5 h, SD = 5.21) samples where PMI was available (t_17=_0.1462, *p* = 0.8855).

### Tissue processing

**Fig. 1:**
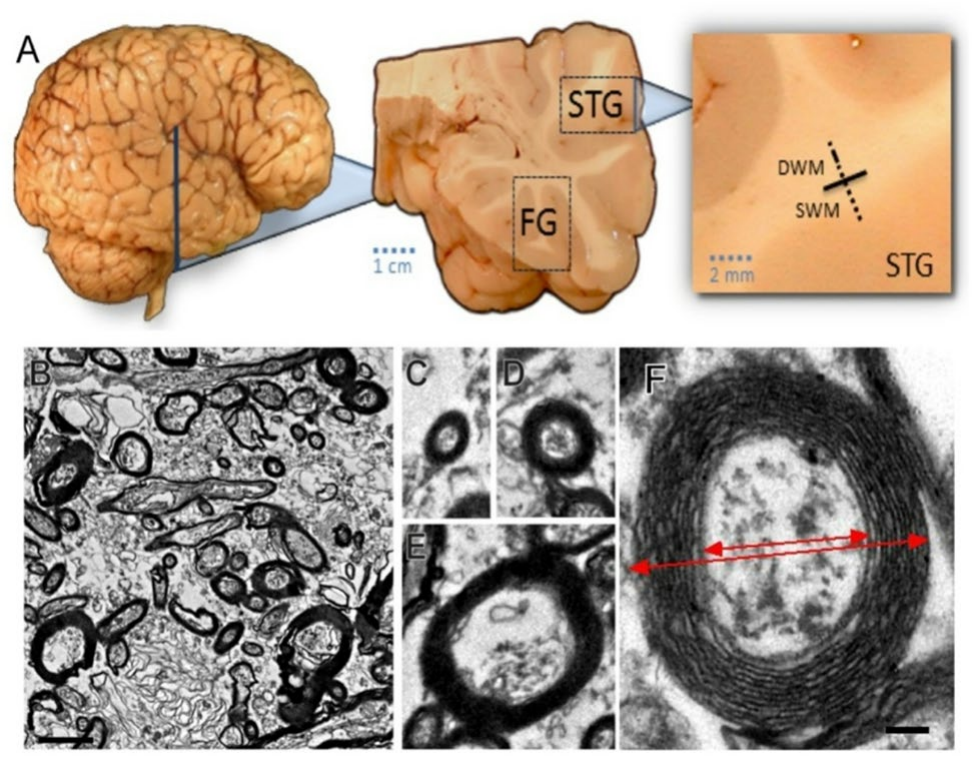
Sampling schema for electron microscopy and representative images of axons by size class. **A** White matter blocks from the superior temporal gyrus (STG) and fusiform gyrus (FG) were sampled. **B** Example photomicrograph of white matter in STG. Scale bar = 2 μm. **C**–**E** Examples of Small, Medium, and Large axons in STG. **F** G-ratio was calculated as the inner diameter divided by the outer axon diameter.

Brain tissue samples were collected from temporal lobe blocks from the right hemisphere of human postmortem brains. Coronal sections were sampled at the level of the mid-rostrocaudal amygdala, as defined previously [63] to ensure consistency across cases. From these sections, white matter deep to the superior temporal sulcus (STS; Brodmann Area 22, temporal association cortex) and the fusiform gyrus (FG) were isolated for analysis (Fig. 1A). Specifically, temporal lobe tissue blocks were cryoprotected using glycerol, sectioned on a freezing microtome at 50 μm, and stored in tissue cryoprotection solution (TCS) at -80 °C. For electron microscopy, a more detailed protocol can be found in Liu and Schumann [64]. Briefly, 50 μm sections were rinsed in 0.1 M phosphate buffer, and the superficial and deep white matter (WM) compartments were excised under a dissecting microscope. SWM was defined as within 2 mm of the gray/white matter boundary, and deep WM (DWM) was defined as from 2 to 4 mm from the gray/white matter boundary (Fig. 1A). Sampling resulted in 4 regions of interest per subject (superficial/deep, STG/FG). The WM dissections were post-fixed in 4% paraformaldehyde/2.5% glutaraldehyde in 0.1 M phosphate buffer, rinsed, and further fixed in 4% aqueous osmium tetroxide (Electron Microscopy Sciences; EMS) for 20 min at room temperature. The samples were then dehydrated in graded ethanol and placed in Araldite solution (Araldite/DMP-30/DDSA; EMS) overnight at 4 °C. The sections were transferred to freshly made Araldite solution for 3 h at room temperature and mounted between siliconized slides and coverslips (Sigmacote). Slides were polymerized for 48–72 h in a 60 °C oven. After polymerization, the sections were examined in the dissecting microscope, the regions of interest were dissected and glued into blank resin blocks and cut at 75 nm using an ultramicrotome (Leica Ultracut). Ultrathin sections were collected onto Formva coated single-slot (1 × 2 mm) copper grids (Ted Pella) and stained using uranyl acetate and lead citrate.

### Imaging and quantification

Imaging was performed on a Philips CM120 Transmission Electron Microscope at 80 kV equipped with a Gatan Bioscan 2K x 2K CCD camera. For density and size distribution analyses, images were acquired at 7400x magnification, covering an area of approximately 156 microns^2^ per image (Fig. 1B). Images were collected blind to diagnosis, with efforts made to acquire images in fields of view where axons appeared to be perpendicularly oriented to the cutting surface to achieve cross-sectional sampling of axon fibers. Ten images were analyzed per subject/region/compartment using NIH ImageJ software (Fiji). Within each image, the diameter of every identified myelinated axon was measured across the center of the axon, or the minor (shortest) axis for ovoid shaped axons. Obliquely sectioned axons (parallel to cut), accounting for less than 10% of total axons, were excluded from quantification due to difficulty in consistently estimating their true diameter to assign to the appropriate category. To minimize inclusion of obliquely sectioned axons, those axons with a major axis judged to be in excess of 1.5 times the diameter of the minor axis were excluded. Incomplete axons at the periphery of images were additionally not included in analyses. To examine differences between local, short range corticocortical and putative long range projection axons [65–68], cluster analyses were used to categorize axons into one of four size categories based on inner axon diameter: small < 0.35 µM, medium 0.36-0.70 µM, large 0.71 − 1.4 µM, and extra large > 1.4 µM (Fig. 1C-F). The breakdown of axons by size class was highly similar to previously published results [21].

For myelin thickness analysis, images were acquired at 8400x covering an area of approximately 39 microns^2^ per image. Thirty images were acquired of single randomly selected axons of sufficient quality. Myelin thickness was measured directly as the difference between inner and outer diameter of axon sheath (Fig. 1F). Myelin thickness was also evaluated using the g-ratio, which is a standardized measure of myelin sheath thickness in proportion to the size of the axon. The g-ratio is expressed as a/A, where a = axon (inner) diameter and A = total (outer) diameter. At least 30 axons were acquired per case/region/compartment of random size but were again classified into size categories based on the above criteria.

### Statistical analysis

Statistical analyses were performed using SAS/STAT software Version 9.4. Linear mixed effect models were used to evaluate both axon density and myelin thickness by axon size (S, M, L, XL), diagnosis (ASD, NT), region (STG, FG) and compartment (SWM, DWM) with all two and three-way interactions and random subject effects to account for within-subject correlation. Planned contrasts compared ASD to NT subjects for each axon size class in each region and compartment. A linear mixed effect model was used to evaluate the relationships between axon density and myelin thickness with age, diagnosis (ASD, NT), region (STG, FG) and compartment (SWM, DWM) with all two and three-way interactions and a random subject effect. From these models, slopes of the relationship between axon density and myelin thickness with age were estimated for ASD and NT subjects in each region and compartment. Planned comparisons of slopes between ASD and NT subjects for each region and compartment were then conducted. For all models, statistically significant three-way interactions were retained and any two-way interactions nested within retained three-way interactions to yield final models. To investigate differences in the relationships of density and myelin thickness with age by size class, we modeled each outcome as a function of age, axon size, and diagnosis separately for each region and compartment combination followed by planned comparisons of slopes between ASD and NT subjects for each size category. Statistical tests were evaluated at a significance level of *α* = 0.05.

## Results

To estimate total axon density, we obtained measures from over 33,400 axons, and counts were distributed evenly by diagnosis (ASD, 16,714; NT, 16,759). Overall, proportions of axons by size were strikingly similar to previous reports from prefrontal cortical regions [21]. The vast majority (> 75%) of axons fell into the small (37.8% ± 4%) or medium (47.3% ± 2.3%) categories in all regions measured and for both ASD and NT subjects. Overall, average axon diameter did not differ between ASD and NT subjects (ASD, 0.51 ± 0.05 µM; NT, 0.54 ± 0.03 µM) and the range of axon diameters was similar between cohorts (ASD, 0.06 to 3.2 µM; NT 0.06 to 3.6 µM) (Supplementary Table S2A).

### ASD associated differences in axon density by size class

**Fig. 2:**
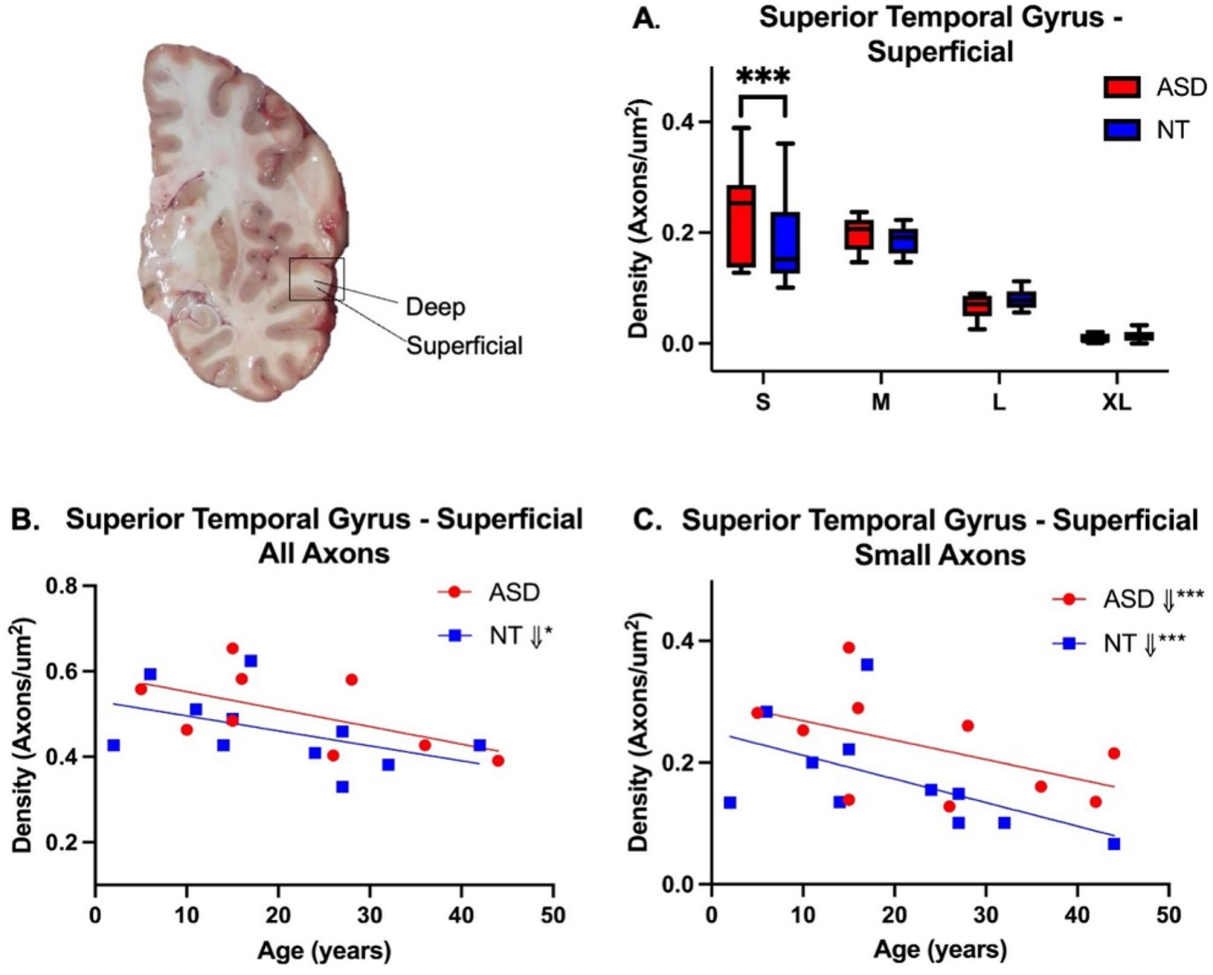
Average axon density in the superficial white matter (SWM) compartment of the superior temporal gyrus (STG) in ASD and NT subjects. A significantly higher density of small axons was observed in ASD cases in STG SWM (**A**). Slopes followed a similar trajectory of decline with age in STG SWM in all axons (**B**) across diagnoses, which was significant in both groups in small axons (**C**)

**Fig. 3:**
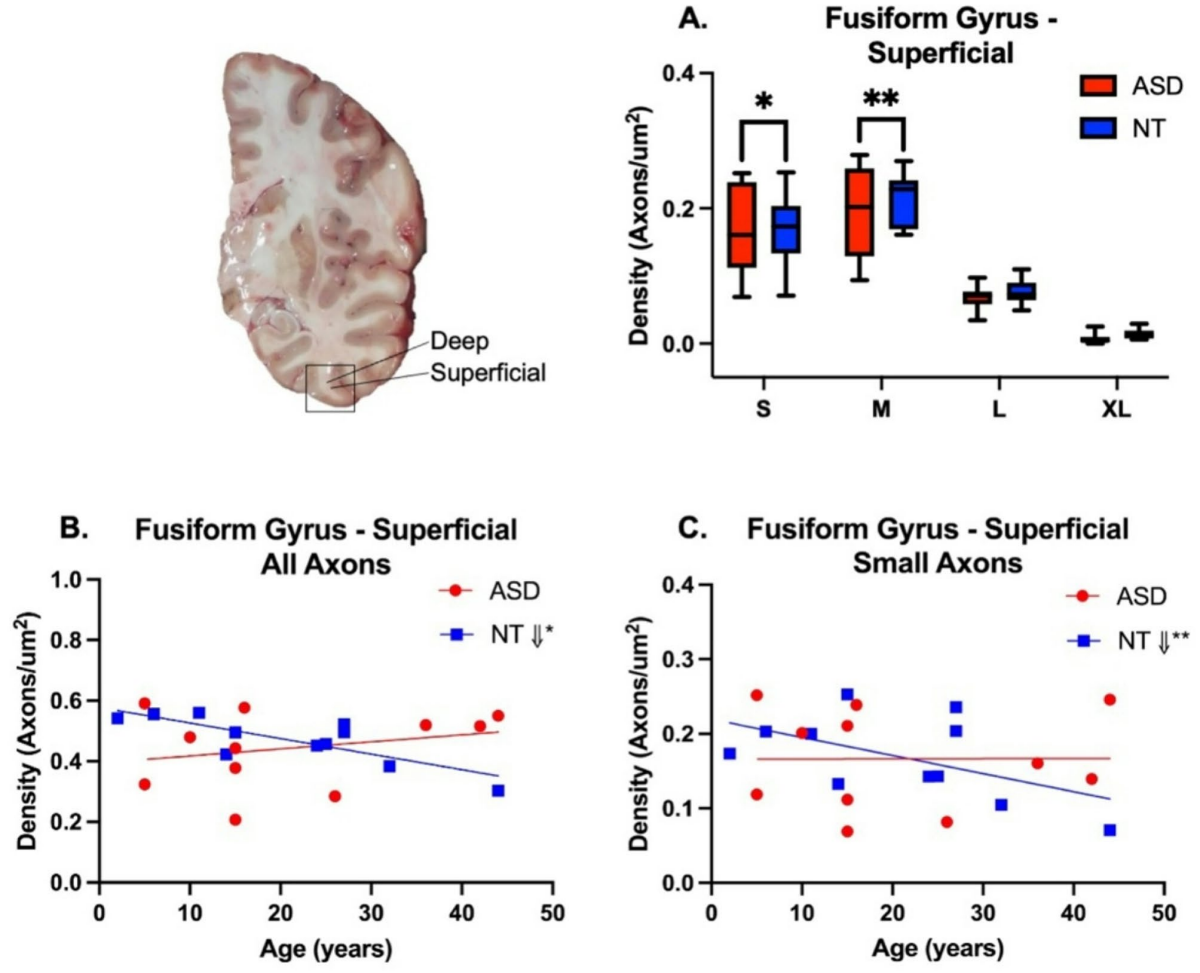
Average axon density in the superficial white matter (SWM) compartment of the fusiform gyrus (FG) in ASD and NT subjects. Small and medium axons were significantly greater in total density in NT subjects (**A**), but a significant difference in slopes of total axon density with age between ASD and NT subjects was observed (**B**, t_22.8_=2.94, *p* = 0.0075). Small axons in FG SWM showed a significant decline with age in NT cases, but not in ASD (**C**)

Linear mixed effect models were used to evaluate axon density versus diagnosis, region, compartment and axon size class (Supplementary Table S3A/B). The density of axons by size class was similar between NT and ASD cases, with a few notable exceptions. In STG SWM, there was a significantly greater density of small caliber axons in ASD relative to NT (ASD, 0.2253 ± 0.09 µM; NT, 0.1843 ± 0.09 µM; F_53.6_=14.70, *p* = 0.0003; Fig. 2A, Supplementary Table S4). Medium-sized axons in STG were also higher in average density in ASD cases, though this difference was not statistically significant (ASD, 0.1980 ± 0.05 µM; NT, 0.1861 ± 0.06 µM; F_53.6_=2.31, *p* = 0.1342). No significant differences were observed in axon density in STG DWM. In FG, there was a general trend for axon densities in ASD to be lower than in NT cases (Fig. 3A) and this difference was significant in small (ASD, 0.1054 ± 0.05 µM; NT, 0.1198 ± 0.07 µM, F_49.6_=5.05, *p* = 0.0292) and medium axons (ASD, 0.1245 ± 0.05 µM; NT, 0.1541 ± 0.07 µM; F_49.6_=10.25, *p* = 0.0024). No differences in axon density were observed in large and XL caliber axons in any region or compartment (Supplementary Table S4).

### Normative postnatal development of white matter axon density

Linear mixed effect models were used to estimate the relationship between total axon density and age by diagnosis, region, and subregion for all subjects (Supplementary Table S5A/B). We observed a significant decline in total axon density from earliest ages examined through middle age in the superficial compartments of both STG (Fig. 2B; t_23.3_=-2.64, *p* = 0.0144) and FG (Fig. 3B; t_23.3_=-2.57, *p* = 0.0170) in NT subjects, which was not observed in the deep compartment (Supplementary Table S6). We next examined the relationship of axon density with age by size class (Supplementary Table S7A/B). NT cases showed significant relationships between axon density and age for small axons in SWM of both regions. Specifically, the density of small axons declined with age in the STG SWM (Fig. 2C). Similarly, small axon densities declined with age in FG SWM (Fig. 3C) and this was also observed in FG DWM (Supplementary Table S8). No age related differences were observed in large and XL caliber axons in any region or compartment.

### Altered postnatal trajectory of white matter axon density in ASD brain

In contrast to NT, total axon density in ASD SWM was not significantly altered with age (Supplementary Table S6). Small axons were higher in density overall in STG SWM, and they showed a similar trend in ASD to NT cases, decreasing in density with advancing age (Fig. 2C, Supplementary Table S8). In contrast to NT, slopes for total axon density in SWM of FG were positive in ASD, and differed significantly from NT where slopes were compared (t_22.8_=2.94, *p* = 0.0075; Supplementary Table S6). Estimated slopes for small and medium axons in FG SWM were also positive, and significantly positive in medium axons (Supplementary Table S8). No significant relationship between density and age was found for small axons in DWM in ASD (Supplementary Figures S1, S2). As in NT individuals, no age related differences were observed in large and XL caliber axon density in any region or compartment in ASD (Supplementary Figures S1, S2). Comparison between ASD and NT groups of the relationship between axon density and age revealed significant differences in slopes in in small axons in deep white matter in STG (t_37.6_=-4.96, *p* < 0.0001) and in small (t_28.9_=2.59, *p* = 0.0149) and medium (t_28.9_=3.97, *p* = 0.0004) axons in SWM of FG (Supplementary Table S8).

### ASDrelated differences in myelin thickness

**Fig. 4:**
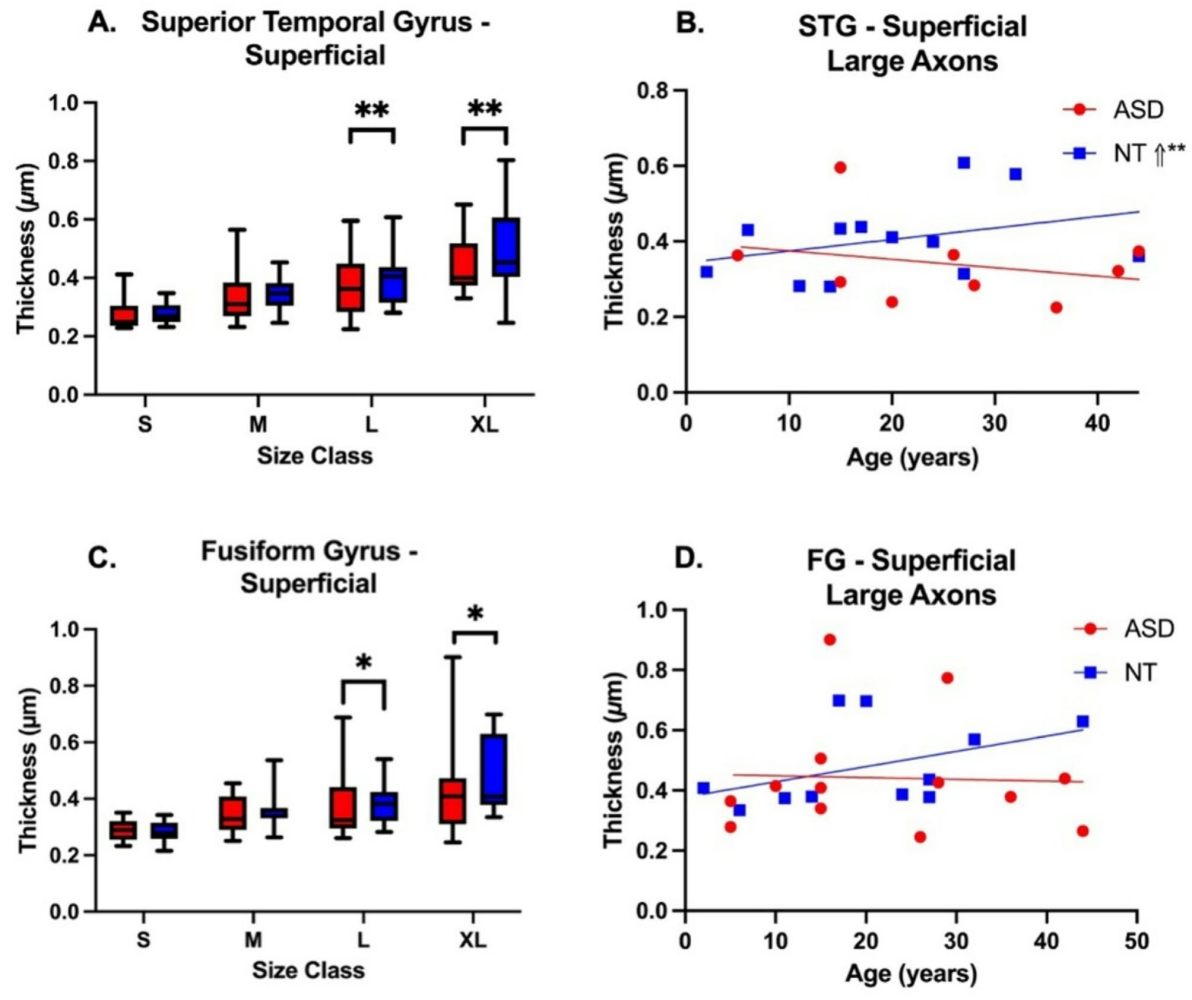
Myelin thickness results for temporal white matter axons in superficial compartments of STG and FG. Left: Mean and 95% confidence intervals for myelin thickness by axon size class; average myelin thickness in large and XL axons was significantly greater in NT than in ASD subjects in both STG (**A**) and FG (**C**). Right: Slopes for NT subjects were positive for NT and negative for ASD subjects in both STG (**B**) and FG (**D**) regions. Comparison of slopes between ASD and NT were significantly different in STG SWM (t_24_=-2.40, *p* = 0.0245, Supplementary Table S13)

A total of 9,397 axons were measured for myelin thickness (Supplementary Table S2B). Axon inner diameters ranged in size from 0.03 µM to approximately 5 µM, while outer diameters (including myelin) ranged between 0.62 µM and 6.5 µM. A broad range of myelin thickness (the difference between outer and inner measures) was also observed, between 0.004 µM and 1.2 µM. Average myelin thickness was consistent across regions and between groups. Small caliber axons displayed the lowest myelin thickness with progressively larger axons having more myelin. Linear mixed effect models were used to evaluate the relationship between myelin thickness versus diagnosis, region, compartment and axon size class (Supplementary Table S9A/B). With regard to diagnosis, no differences in axonal myelin thickness were observed for small and medium caliber axons in any region or area examined. Likewise, no significant difference in myelin thickness was observed in DWM for STG and FG between ASD and NT. However, in SWM of FG and STG, average myelin thickness was significantly decreased for both large and extra-large caliber axons in ASD cases (Fig. 4; Supplementary Table S10).

To account for myelin thickness relative to axon size, we also calculated the g-Ratio, or the fraction of inner axon diameter to total outer diameter (axon plus myelin), which has been taken as a measure of efficiency of axon conduction [69]. To determine if altered myelin thickness could be indicative of altered large scale axonal conduction we measured average g-ratios in all regions (Supplementary Figure S4). Indeed, previous studies had suggested g-ratios decreased in ASD as a result of altered densities of thinly myelinated axons [20, 21]. However, when we compared average g-ratios in ASD and NT groups we found no significant differences (Supplementary Table S11).

### Altered postnatal trajectory of myelin thickness in ASD

Linear mixed effect models were used to evaluate the relationship of myelin thickness with age by diagnosis, region, and compartment (Supplementary Table S12A/B). For the relationship between myelin thickness and age, comparison of estimated slopes showed opposing developmental trends of increased myelin thickness with age in NT, and decreased myelin thickness in ASD in STG SWM (Fig. 3C, D; Supplementary Table S13). ASD and NT trajectories differed significantly in STG SWM (t_24_=-2.40, *p* = 0.0245), suggesting normative developmental increases in myelin thickness were not observed in ASD.

We next sought to determine if altered myelin thickness was associated with a particular axon size class (Supplementary Table S14A/B, Supplementary Figure S3). Notably, all estimates of slope were positive for NT subjects, however, negative slopes were observed for Large and XL axons in ASD in STG SWM (Fig. 4B), as well as large axons in both compartments of FG (Supplementary Table S15; Fig. 4D). A significant difference in slopes was observed for myelin thickness in large axons in SWM between NT and ASD (t_26.6_=-3.78, *p* = 0.0008). Thus, larger axons in SWM of STG did not exhibit the same developmental increase in myelin thickness seen in NTs.

## Discussion

**Fig. 5:**
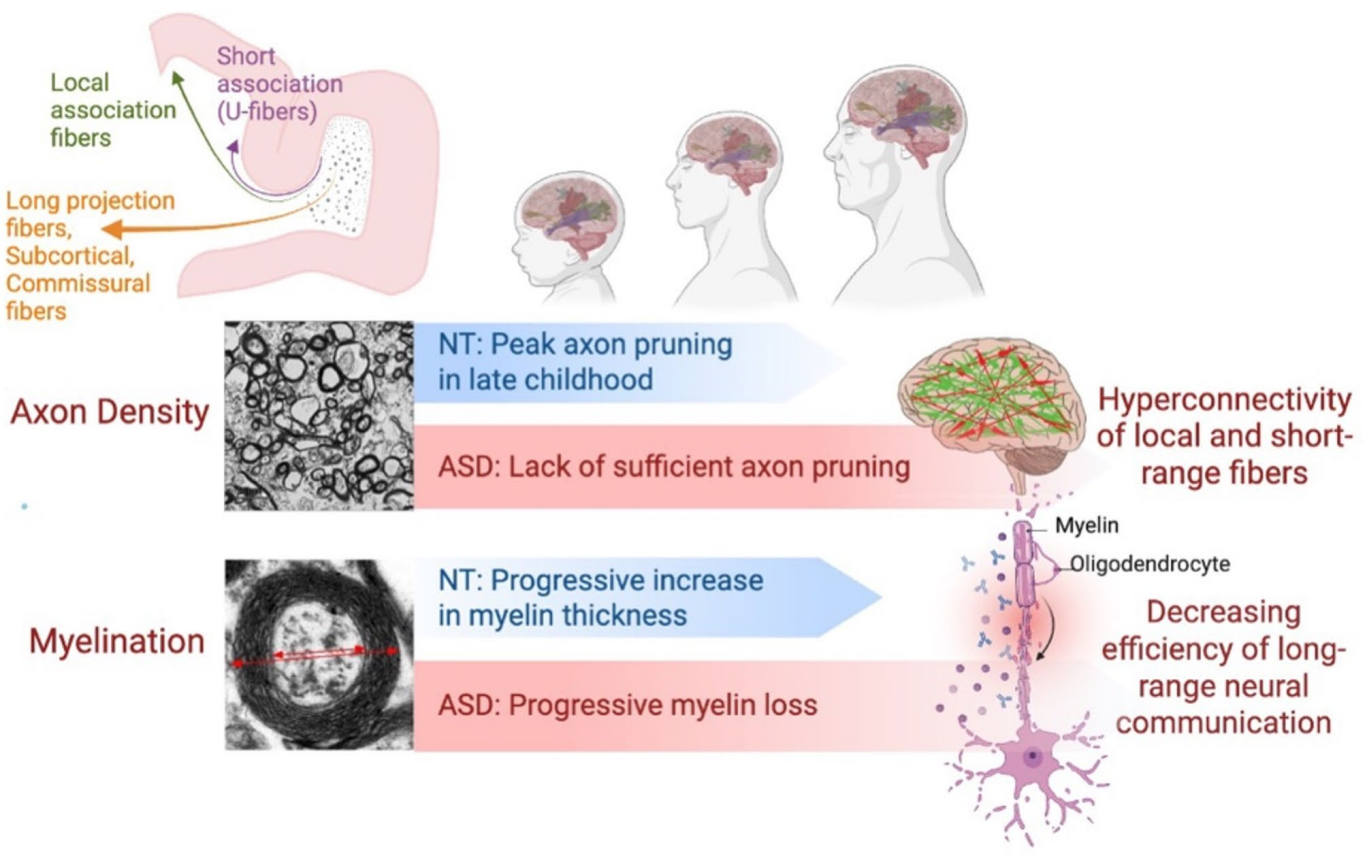
Axonal pruning and myelin development is altered in ASD. Whereas late childhood is a peak period for the pruning of small axons in the neurotypical (NT) brain, we have demonstrated that the temporal lobe in ASD is characterized by a persistent abundance of small axons, suggesting aberrant pruning in superficial layers which may contribute to local hyperconnectivity, and alter the balance of local vs. long-ranging neuronal signaling. While myelin thickness increases with age on large axons in NT cases, this was not observed in superficial white matter in the temporal lobe in ASD, which may show a pattern of decreasing myelin thickness with age. These alterations to typical white matter development may contribute to worsening symptomatology and cognitive decline with age in ASD. Illustration created with BioRender

Temporal lobe white matter in males with ASD follows a developmental trajectory distinct from that of neurotypical males, generally characterized by increased axon density and reduced myelin thickness across postnatal development. In neurotypical individuals, axon pruning and progressive myelination refine white matter tracts to support efficient communication [2, 6, 70–73]. In ASD, small-diameter axons remain abundant in the superficial white matter of the superior temporal gyrus, and age-related pruning of axon density is diminished in the fusiform gyrus (FG). Myelin sheaths around large-diameter axons in ASD do not follow the typical developmental pattern of thickening with age, resulting in persistently thinner myelin. In neurotypical development, myelination of large axons in superficial white matter likely facilitates communication between adjacent associative cortical regions, while large-diameter axons in deep white matter support long-range connections between cortical and subcortical areas [74] (Fig. 5). The absence of group differences in myelin thickness or axon density in STG DWM suggests that long-range pathways may remain relatively spared in ASD, whereas structural differences in SWM implicate local connectivity as a more vulnerable target. These findings are consistent with functional imaging studies reporting disrupted local—but not global—network dynamics in ASD, particularly within temporal association regions [75]. Disruptions in the integration and timing of local signal propagation may contribute to core behavioral features of ASD, including challenges with language processing, social reciprocity, and sensory integration—functions that depend on coordinated activity within temporal lobe circuits early in development. These early alterations may set the stage for persistent differences in network organization and function across the lifespan.

Alterations in temporal lobe white matter in ASD strongly parallel patterns previously identified with similar EM approaches in the postmortem prefrontal cortex (PFC). Specifically, Zikopoulos and Barbas (2010) examined three PFC regions, including the anterior cingulate cortex (ACC; BA 32), orbitofrontal cortex (OFC; BA 11), and dorsolateral prefrontal cortex (DLPFC; BA 46). In the ACC, they observed a significant increase in the density of small axons, particularly significant in the superficial compartment, along with a reduction in the density of XL axons in ASD. Average myelin thickness was lower overall in the ACC, though this was attributed to the predominance of small axons as compared to larger, more thickly myelinated axons. In the OFC, myelin thickness was significantly reduced in ASD, irrespective of axonal diameter. Laminar thickness and neuron density did not differ in the ACC, leading the authors to attribute axon density differences to increased axonal branching, which was further supported by elevated GAP-43 expression differences in the ACC of ASD cases, but not in other PFC regions. To assess age-related changes in white matter microstructure, Zikopoulos and colleagues (2018) further examined the ACC in a larger postmortem sample. In neurotypical cases, the density of small and medium axons remains relatively stable with age, whereas in ASD, increases were observed, particularly in superficial white matter. Additionally, the density of thick, long-range axons decreased with age in the ACC in ASD, whereas they increased with age in controls. These findings support the hypothesis that enhanced short-range connectivity, and reduced longer-ranging connections, may be a key feature of neural communication in ASD [54, 76, 77]. Such divergent developmental trajectories likely contribute to imbalances in cortical signaling, particularly across the frontal and temporal cortices and their shared networks [19].

This is the first study to directly examine axonal alterations in temporal lobe white matter ultrastructure in ASD. Our findings indicate that trade-offs between local and global connectivity, consistent with the patterning of short- and long-ranging axons [78–80], extend beyond the prefrontal cortex. Although prefrontal circuitry is strongly affected in ASD, the observed temporal lobe changes highlight that disrupted white matter architecture is not confined to frontal regions. Instead, these alterations add to converging evidence implicating temporal lobe circuits in the social cognitive and emotional differences associated with ASD. Within temporal cortices, the superior temporal sulcus, fusiform gyrus, and amygdala are consistently implicated in ASD neuropathology across functional, structural, cellular, and molecular levels [81]. For instance, the superior temporal gyrus in toddlers with ASD is enlarged by approximately 10% compared to typical development [61]. Additionally, the amygdala is enlarged in children with ASD, with an increased number of neurons and dendritic spines relative to neurotypical children [82–85] followed by a loss of neurons and spine density in adulthood [85, 86]. Individuals with ASD also display reduced density and soma size of neurons in the fusiform gyrus [87]. Changes in cell numbers, connectivity, and organization would have significant implications for the axonal projections emerging from these regions, contributing to the atypical patterns of axon density and myelination we have observed in temporal white matter in ASD.

Consistent with these findings, neuroimaging studies report persistent alterations in white matter microstructure across the lifespan in ASD [88] which may correlate with symptom severity [89]. DTI studies associate ASD symptoms with disruptions in connectivity and white matter structure, linking these changes to impairments in communication and cognitive function [15, 33–36, 90–92]. Fractional anisotropy, which reflects the directionality of water diffusion in white matter tracts, tends to be elevated young ASD subjects [49, 89, 93, 94] but decreases in adolescence and adulthood, suggesting a postnatal developmental shift [29, 33, 36, 38–46, 48, 59, 95–97]. These trajectories point to altered structural maturation processes involving axon diameter, density, and/or myelination—factors likely to be broadly dysregulated in ASD [98]. Accelerated cortical thinning in the temporal lobe has also been noted in adults with ASD relative to neurotypical peers [99] and is associated with executive function impairments during the transition to adulthood [100].

Our findings indicate that the myelin sheath surrounding large-diameter axons in superficial white matter adjacent to STG does not follow the normative pattern of progressive thickening with age in individuals with ASD, pointing to fundamental deviations in myelin structure and maturation. This disruption may reflect broader mechanisms affecting oligodendrocyte function and myelin development. Indeed, animal models that reflect phenotypes found in syndromic forms of ASD show significant alterations in myelination and oligodendrocyte function [101–106]. Transcriptomic data from human ASD cases also show dysregulation of genes associated with oligodendrocyte biology and myelin-associated pathways [107, 108]. Moreover, adults with ASD have significantly fewer oligodendrocytes in the amygdala compared to age-matched controls, suggesting age-related loss that may contribute to myelin thinning [109]. Thus, disrupted myelin development and oligodendrocyte function may be convergent features of both syndromic and non-syndromic ASD. Diverse genetic and molecular etiologies may lead to a common outcome of altered white matter integrity, contributing to circuit-level changes in connectivity and behavioral phenotypes characteristic of ASD.

Alterations in axon and myelin development are not unique to autism. Many psychiatric disorders involve changes in white matter microstructure and myelination [110–112] including schizophrenia [113, 114], bipolar disorder [115] and major depression [116]. Disruptions in white matter integrity have been shown to correlate with clinical evidence of increasing symptom severity in schizophrenia [117], and these changes may precede the onset of psychotic symptoms [118]. Changes in myelin content and indicators of intra-axonal integrity associated with impaired signal transduction may further contribute to cognitive deficits seen in schizophrenia [119]. Broad dysregulation of genes associated with oligodendrocytes and oligodendrocyte precursor cells has been noted across psychiatric disorders [120–123] including schizophrenia [121, 124, 125]. Early-life and chronic psychosocial stress can contribute to variation in white matter development [126–129], potentially highlighting periods of vulnerability in childhood and adolescence. Overall, white matter and myelin development involve dynamic processes across the lifespan, and understanding patterns of change in axonal microstructure may highlight windows of opportunity for therapeutic interventions in neurodevelopmental and psychiatric conditions.

### Limitations

Limitations inherent to human postmortem research, including small sample sizes, variable tissue quality, minimal demographic information, and cross-sectional design, may obscure developmental trajectories and limit correlating symptomatology with underlying biological processes. Despite these challenges, we detected significant and region-specific alterations in axon density and myelin thickness in ASD. Additionally, myelin thickness and axon density were examined separately in control and ASD subjects. Given that axon density and myelin thickness are likely correlated, future designs may include multivariate models to further understand how white matter architecture changes in the temporal lobe with age in neurotypical individuals and those with neurodevelopmental and psychiatric disorders. Further, although our study focused on males due to limited tissue availability from females, ongoing work should continue to investigate sex differences in white matter maturation across the lifespan [61, 130–135].

## Conclusions

Our findings reveal structural changes in axonal microstructure in ASD consistent with a developmental pattern of local hyperconnectivity—driven by increased density of small axons—and reduced integrative connectivity, reflected in thinner myelin on large-caliber axons in superficial white matter. Although little is known about brain aging in ASD, our results align with growing evidence of continued neuroanatomical changes into adulthood, including regional volume loss [136–141], cortical thinning [99, 100, 142–147], and altered white matter integrity [20, 88, 89, 148–153]. Future studies may clarify whether neuroinflammatory signaling influences oligodendrocyte maturation or axon–glia interactions, contributing to the white matter changes observed in ASD [154]. Together, these findings underscore the importance of understanding how disrupted white matter maturation affects neural communication and functional outcomes. Continued research into the genetic, molecular, and environmental factors shaping these trajectories is essential to identify critical windows for intervention across the lifespan.

## Supplementary Information

Below is the link to the electronic supplementary material.

Supplementary Material 1

Supplementary Material 2

## Data Availability

No datasets were generated or analysed during the current study.
