# Supplementary material for: Age-related differences in axon pruning and myelination may alter neural signaling in autism spectrum disorder
Source: Mol Autism. 2025 Oct 23;16:53. doi: 10.1186/s13229-025-00684-y (PMC12548170; doi:10.1186/s13229-025-00684-y)
Supplement: Supplementary file 1 — Supplementary Material 1 [file 13229_2025_684_MOESM1_ESM.docx]

SUPPLEMENTARY INFORMATION.

**Supplementary Table S1**. Demographic information for cases used in this study.

| Case ID | Diagnosis | Age | Sex | PMI |
| --- | --- | --- | --- | --- |
| H-3-03 | ASD | 5 | M | 15.5 |
| H-1-03 | ASD | 5 | M | **--** |
| H-11-01 | ASD | 10 | M | 24 |
| H-10-03 | ASD | 15 | M | **--** |
| H-5-02 | ASD | 15 | M | 4 |
| H-4-99 | ASD | 15 | M | **--** |
| H-15-01 | ASD | 16 | M | 48 |
| H-10-01 | ASD | 20 | M | 23.6 |
| H-2-01 | ASD | 26 | M | 8.3 |
| H-6-04 | ASD | 28 | M | 18 |
| H-2-02 | ASD | 29 | M | 20 |
| H-3-04 | ASD | 36 | M | 24 |
| H-7-02 | ASD | 42 | M | 24 |
| H-6-00 | ASD | 44 | M | 31 |
| **ASD Average** |  | 21.86 |  | 21.85 |
| **ASD SD** |  | 12.68 |  | 11.60 |
| H-2-03 | NT | 2 | M | -- |
| H-3-02 | NT | 6 | M | 18 |
| H-2-04 | NT | 11 | M | 30 |
| H-8-02 | NT | 14 | M | 19 |
| H-16-01 | NT | 15 | M | 21 |
| H-9-02 | NT | 17 | M | -- |
| H-4-98 | NT | 20 | M | 26 |
| H-6-02 | NT | 24 | M | 29 |
| H-8-03 | NT | 25 | M | -- |
| H-13-01 | NT | 27 | M | 21 |
| H-11-02 | NT | 27 | M | 16 |
| H-4-03 | NT | 32 | M | -- |
| H-19-01 | NT | 44 | M | -- |
| **NT Average** |  | 20.31 |  | 22.5 |
| **NT SD** |  | 11.29 |  | 5.21 |

**Supplementary Table S2A.** Distribution of axons by size class in temporal cortex. Values for prefrontal cortex (BA 32, 11, and 46) are drawn from Zikopoulos et al., 2010, showing similarity in axon distribution by size class across regions and diagnoses.

| **Region** | **WM Region** | **Dx** | **Number Sampled** | **S (%)** | **M (%)** | **L (%)** | **XL (%)** | **Avg Size (µM)** | **Min (µM)** | **Max (µM)** |
| --- | --- | --- | --- | --- | --- | --- | --- | --- | --- | --- |
| STG | DWM | ASD | 4179 | 38.5 | 45.2 | 13.3 | 3 | 0.53 | 0.03 | 3.3 |
|  |  | NT | 3789 | 37 | 46 | 13 | 3.8 | 0.55 | 0.03 | 3.9 |
|  | SWM | ASD | 4696 | 45.7 | 44.7 | 8.2 | 1.4 | 0.46 | 0.05 | 3.2 |
|  |  | NT | 4263 | 40.5 | 45.8 | 11.6 | 2.2 | 0.51 | 0.06 | 2.7 |
| FG | DWM | ASD | 3205 | 34 | 47 | 14.2 | 4.3 | 0.57 | 0.09 | 3.3 |
|  |  | NT | 4096 | 32.5 | 48.3 | 14.5 | 4.7 | 0.58 | 0.09 | 3.9 |
|  | SWM | ASD | 4634 | 38 | 50.6 | 10.1 | 1.3 | 0.49 | 0.06 | 2.8 |
|  |  | NT | 4611 | 36.3 | 50.6 | 10.8 | 2.2 | 0.52 | 0.05 | 3.8 |
| *BA 32* | *DWM* | *ASD* |  | *30* | *50* | *18* | *2* | *0.53* |  | |
|  |  | *NT* |  | *29* | *49* | *16* | *7* | *0.58* |  |  |
|  | *SWM* | *ASD* |  | *44* | *43* | *11* | *1* | *0.45* |  |  |
|  |  | *NT* |  | *31* | *49* | *16* | *4* | *0.55* |  |  |
| *BA 11* | *DWM* | *ASD* |  | *29* | *49* | *20* | *2* | *0.53* |  |  |
|  |  | *NT* |  | *38* | *46* | *13* | *2* | *0.49* |  |  |
|  | *SWM* | *ASD* |  | *43* | *44* | *12* | *1* | *0.44* |  |  |
|  |  | *NT* |  | *46* | *43* | *10* | *4* | *0.44* |  |  |
| *BA 46* | *DWM* | *ASD* |  | *38* | *42* | *16* | *5* | *0.52* |  |  |
|  |  | *NT* |  | *36* | *42* | *18* | *3* | *0.53* |  |  |
|  | *SWM* | *ASD* |  | *36* | *47* | *16* | *1* | *0.50* |  |  |
|  |  | *NT* |  | *36* | *42* | *19* | *4* | *0.54* |  |  |

**Supplementary Table S2B.** Total number of axons measured for myelin thickness by region, compartment, and diagnosis.

| **Region** | **Compartment** | **Diagnosis** | **Axons** |
| --- | --- | --- | --- |
| **STG** | **SWM** | ASD | 988 |
|  |  | NT | 1,109 |
|  |  | Total | 2,097 |
|  | **DWM** | ASD | 1,771 |
|  |  | NT | 947 |
|  |  | Total | 2,718 |
| **FG** | **SWM** | ASD | 1,603 |
|  |  | NT | 1,041 |
|  |  | Total | 2,644 |
|  | **DWM** | ASD | 872 |
|  |  | NT | 1,066 |
|  |  | Total | 1,938 |
| All Regions | All Compartments | Total Axons Measured | 9,397 |

**Supplementary Tables 3A-B.** Full results of linear mixed effect model evaluating axon density versus diagnosis, region, compartment and axon size class.

**Supplementary Table S3A.** Type 3 statistics from linear mixed effect model evaluating axon density versus diagnosis (Dx), region (Region), compartment (Comp), axon size class (Size). The 3-way interaction Region*Comp*Dx was not statistically significant (p= 0.49) and was not retained in the final model shown here.

| Effect | Num DF | Den DF | F Value | Pr > F |
| --- | --- | --- | --- | --- |
| Dx | 1 | 22.9 | 0.14 | 0.7097 |
| Region | 1 | 3452 | 23.20 | <.0001 |
| Region*Dx | 1 | 3452 | 26.51 | <.0001 |
| Comp | 1 | 3452 | 169.96 | <.0001 |
| Comp*Dx | 1 | 3452 | 9.43 | 0.0022 |
| Region*Comp | 1 | 3452 | 19.07 | <.0001 |
| Size | 3 | 3427 | 2160.27 | <.0001 |
| Dx*Size | 3 | 3427 | 7.44 | <.0001 |
| Region*Size | 3 | 3427 | 26.99 | <.0001 |
| Comp*Size | 3 | 3427 | 87.71 | <.0001 |
| Region*Comp*Size | 3 | 3427 | 6.67 | 0.0002 |
| Region*Dx*Size | 3 | 3427 | 7.03 | 0.0001 |
| Comp*Dx*Size | 3 | 3427 | 6.17 | 0.0004 |

**Supplementary Table S3B.** Parameter estimates for effect of diagnosis, region, white matter component and size class on axon density from a linear mixed effect model.

| **Effect** | **Region** | **Comp** | **Dx** | **Size** | **Estimate** | **SE** | **DF** | **T** | **P** |
| --- | --- | --- | --- | --- | --- | --- | --- | --- | --- |
| **Intercept** |  |  |  |  | 0.009612 | 0.006828 | 56.5 | 1.41 | 0.1647 |
| **Dx** |  |  | ASD |  | -0.00473 | 0.009459 | 52 | -0.50 | 0.6194 |
| **Region** | FG |  |  |  | 0.004492 | 0.005598 | 3432 | 0.80 | 0.4223 |
| **Region*Dx** | FG |  | ASD |  | -0.00344 | 0.006514 | 3437 | -0.53 | 0.5979 |
| **Comp** |  | D |  |  | 0.006080 | 0.005698 | 3433 | 1.07 | 0.2860 |
| **Comp*Dx** |  | D | ASD |  | 0.002602 | 0.006510 | 3437 | 0.40 | 0.6894 |
| **Region*Comp** | FG | D |  |  | 0.000969 | 0.006530 | 3439 | 0.15 | 0.8820 |
| **Size** |  |  |  | L | 0.06383 | 0.006011 | 3427 | 10.62 | <.0001 |
| **Size** |  |  |  | M | 0.1707 | 0.006011 | 3427 | 28.41 | <.0001 |
| **Size** |  |  |  | S | 0.1637 | 0.006011 | 3427 | 27.24 | <.0001 |
| **Dx*Size** |  |  | ASD | L | -0.00474 | 0.008009 | 3427 | -0.59 | 0.5537 |
| **Dx*Size** |  |  | ASD | M | 0.01874 | 0.008009 | 3427 | 2.34 | 0.0194 |
| **Dx*Size** |  |  | ASD | S | 0.04802 | 0.008009 | 3427 | 6.00 | <.0001 |
| **Region*Size** | FG |  |  | L | 0.001575 | 0.007881 | 3427 | 0.20 | 0.8417 |
| **Region*Size** | FG |  |  | M | 0.02525 | 0.007881 | 3427 | 3.20 | 0.0014 |
| **Region*Size** | FG |  |  | S | -0.01323 | 0.007881 | 3427 | -1.68 | 0.0933 |
| **Comp*Size** |  | D |  | L | -0.00192 | 0.008009 | 3427 | -0.24 | 0.8105 |
| **Comp*Size** |  | D |  | M | -0.02378 | 0.008009 | 3427 | -2.97 | 0.0030 |
| **Comp*Size** |  | D |  | S | -0.03565 | 0.008009 | 3427 | -4.45 | <.0001 |
| **Region*Comp*Size** | FG | D |  | L | -0.01123 | 0.009144 | 3427 | -1.23 | 0.2195 |
| **Region*Comp*Size** | FG | D |  | M | -0.03929 | 0.009144 | 3427 | -4.30 | <.0001 |
| **Region*Comp*Size** | FG | D |  | S | -0.01203 | 0.009144 | 3427 | -1.32 | 0.1883 |
| **Region*Dx*Size** | FG |  | ASD | L | 0.001016 | 0.009144 | 3427 | 0.11 | 0.9115 |
| **Region*Dx*Size** | FG |  | ASD | M | -0.02184 | 0.009144 | 3427 | -2.39 | 0.0170 |
| **Region*Dx*Size** | FG |  | ASD | S | -0.03399 | 0.009144 | 3427 | -3.72 | 0.0002 |
| **Comp*Dx*Size** |  | D | ASD | L | 0.001108 | 0.009139 | 3427 | 0.12 | 0.9035 |
| **Comp*Dx*Size** |  | D | ASD | M | -0.02086 | 0.009139 | 3427 | -2.28 | 0.0225 |
| **Comp*Dx*Size** |  | D | ASD | S | -0.03152 | 0.009139 | 3427 | -3.45 | 0.0006 |

**Supplementary Table S4.** Results of planned contrasts evaluating differences in axon density between ASD and NT by size class in each brain region and white matter type. For all analyses, p-values <0.05 were taken as statistically significant; *indicates p<0.05, **p<0.01, ***p<0.001.

| Region | Num DF | Den DF | F Value | Pr > F |
| --- | --- | --- | --- | --- |
| Large ASD vs. NT for DWM In STG | 1 | 51.8 | 0.34 | 0.5645 |
| Medium ASD vs. NT for DWM In STG | 1 | 51.8 | 0.19 | 0.6625 |
| Small ASD vs. NT for DWM In STG | 1 | 51.8 | 1.62 | 0.2081 |
| XL ASD vs. NT for DWM In STG | 1 | 51.8 | 0.03 | 0.8722 |
| Large ASD vs. NT for SWM In STG | 1 | 53.6 | 0.82 | 0.3688 |
| Medium ASD vs. NT for SWM In STG | 1 | 53.6 | 2.31 | 0.1342 |
| **Small ASD vs. NT for SWM In STG** | **1** | **53.6** | **14.70** | **0.0003***** |
| XL ASD vs. NT for SWM In STG | 1 | 53.6 | 0.10 | 0.7502 |
| Large ASD vs. NT for DWM In FG | 1 | 49.6 | 0.84 | 0.3647 |
| **Medium ASD vs. NT for DWM In FG** | **1** | **49.6** | **10.25** | **0.0024**** |
| **Small ASD vs. NT for DWM In FG** | **1** | **49.6** | **5.05** | **0.0292*** |
| XL ASD vs. NT for DWM In FG | 1 | 49.6 | 0.43 | 0.5128 |
| Large ASD vs. NT for SWM In FG | 1 | 50.5 | 1.56 | 0.2178 |
| Medium ASD vs. NT for SWM In FG | 1 | 50.5 | 1.44 | 0.2357 |
| Small ASD vs. NT for SWM In FG | 1 | 50.5 | 0.14 | 0.7081 |
| XL ASD vs. NT for SWM In FG | 1 | 50.5 | 0.67 | 0.4168 |

**Supplementary Tables S5A-B.** Full results of linear mixed effect model evaluating axon density versus age by by diagnosis, region and compartment.

**Supplementary Table S5A.** Results of final model evaluating the relationship between axon density and age by diagnosis, region and subregion. A linear mixed effect model was used to evaluate the relationship between axon density and age by diagnosis, region and subregion with all two and three-way interactions. The three-way interaction of diagnosis*region*subregion (p = 0.656) was not statistically significant and was dropped from the model.

| **Effect** | **Num DF** | **Den DF** | **F Value** | **Pr > F** |
| --- | --- | --- | --- | --- |
| **Age** | 1 | 20.6 | 1.99 | 0.1728 |
| **Dx** | 1 | 20.6 | 1.44 | 0.2435 |
| **Age*Dx** | 1 | 20.6 | 1.52 | 0.2308 |
| **Region** | 1 | 826 | 23.01 | <.0001 |
| **Age*Region** | 1 | 825 | 3.04 | 0.0817 |
| **Dx*Region** | 1 | 825 | 66.69 | <.0001 |
| **Comp** | 1 | 826 | 129.09 | <.0001 |
| **Age*Comp** | 1 | 826 | 15.27 | 0.0001 |
| **Dx*Comp** | 1 | 826 | 2.74 | 0.0980 |
| **Region*Comp** | 1 | 826 | 1.45 | 0.2295 |
| **Age*Dx*Region** | 1 | 824 | 30.76 | <.0001 |
| **Age*Dx*Comp** | 1 | 826 | 20.12 | <.0001 |
| **Age*Region*Comp** | 1 | 826 | 21.08 | <.0001 |

**Supplementary Table 5B.** Parameter estimates for the final model relating axon density to age, diagnosis, region and subregion.

| **Effect** | **Dx** | **Region** | **Comp** | **Estimate** | **Standard Error** | **DF** | **t Value** | **Pr > \|t\|** |
| --- | --- | --- | --- | --- | --- | --- | --- | --- |
| **Intercept** |  |  |  | 0.5485 | 0.04710 | 24.6 | 11.64 | <.0001 |
| **Age** |  |  |  | -0.00556 | 0.002081 | 26.3 | -2.67 | 0.0128 |
| **Dx** | ASD |  |  | -0.00360 | 0.06397 | 24 | -0.06 | 0.9556 |
| **Age*Dx** | ASD |  |  | 0.002723 | 0.002700 | 24.3 | 1.01 | 0.3232 |
| **Region** |  | FG |  | 0.02331 | 0.01949 | 825 | 1.20 | 0.2321 |
| **Age*Region** |  | FG |  | 0.000341 | 0.000889 | 826 | 0.38 | 0.7014 |
| **Dx*Region** | ASD | FG |  | -0.1765 | 0.02161 | 825 | -8.17 | <.0001 |
| **Comp** |  |  | D | -0.1541 | 0.01938 | 825 | -7.95 | <.0001 |
| **Age*Comp** |  |  | D | 0.006013 | 0.000912 | 829 | 6.59 | <.0001 |
| **Dx*Comp** | ASD |  | D | 0.03588 | 0.02166 | 826 | 1.66 | 0.0980 |
| **Region*Comp** |  | FG | D | 0.02583 | 0.02148 | 826 | 1.20 | 0.2295 |
| **Age*Dx*Region** | ASD | FG |  | 0.005135 | 0.000926 | 824 | 5.55 | <.0001 |
| **Age*Dx*Comp** | ASD |  | D | -0.00418 | 0.000932 | 826 | -4.49 | <.0001 |
| **Age*Region*Comp** |  | FG | D | -0.00420 | 0.000915 | 826 | -4.59 | <.0001 |

**Supplementary Table S6.** Estimates of the relationship between total axon density and age for NT and ASD subjects in STG and FG. A significant decline with age was observed in total axon density in the superficial compartment in both temporal regions in NT individuals, which was not significant in ASD STG SWM, and was positive, but not significant in ASD FG SWM.

| **Label** | **Estimate** | **SE** | **DF** | **t Value** | **Pr > \|t\|** | **Lower** | **Upper** |
| --- | --- | --- | --- | --- | --- | --- | --- |
| **NT STG SWM** | **-0.00533** | **0.002015** | **23.3** | **-2.64** | **0.0144*** | **-0.00949** | **-0.00116** |
| NT STG DWM | 0.000487 | 0.002025 | 23.8 | 0.24 | 0.8119 | -0.00369 | 0.004669 |
| **NT FG SWM** | **-0.00518** | **0.002014** | **23.3** | **-2.57** | **0.0170*** | **-0.00934** | **-0.00101** |
| NT FG DWM | -0.00344 | 0.002015 | 23.3 | -1.71 | 0.1009 | -0.00761 | 0.000723 |
| ASD STG SWM | -0.00280 | 0.001767 | 24.4 | -1.59 | 0.1255 | -0.00645 | 0.000841 |
| ASD STG DWM | -0.00103 | 0.001752 | 23.6 | -0.59 | 0.5614 | -0.00465 | 0.002587 |
| ASD FG SWM | 0.002613 | 0.001752 | 23.6 | 1.49 | 0.1490 | -0.00101 | 0.006232 |
| ASD FG DWM | 0.000304 | 0.001754 | 23.7 | 0.17 | 0.8640 | -0.00332 | 0.003926 |
| STG SWM: ASD vs. NT | 0.002526 | 0.002660 | 23.1 | 0.95 | 0.3522 | -0.00298 | 0.008027 |
| STG DWM: ASD vs. NT | -0.00152 | 0.002659 | 23.0 | -0.57 | 0.5733 | -0.00702 | 0.003980 |
| **FG SWM: ASD vs. NT** | **0.007790** | **0.002653** | **22.8** | **2.94** | **0.0075**** | **0.002299** | **0.01328** |
| FG DWM: ASD vs. NT | 0.003745 | 0.002655 | 22.9 | 1.41 | 0.1718 | -0.00175 | 0.009239 |

**Supplementary Tables 7A-B.** Results from linear mixed effect models relating axon density to age, axon size and diagnosis for each region and compartment.

**Supplementary Table S7A.** Type 3 statistics from linear mixed effect model evaluating axon density versus diagnosis (Dx), region (Region), compartment (Comp), axon size class (Size). The 3-way interaction Region*Comp*Dx was not statistically significant (p= 0.49) and was not retained in the final model shown here.

| **Effect** | **Num DF** | **Den DF** | **F Value** | **Pr > F** |
| --- | --- | --- | --- | --- |
| **Age** | 1 | 20.5 | 1.94 | 0.1790 |
| **Dx** | 1 | 20.6 | 1.41 | 0.2482 |
| **Age*Dx** | 1 | 20.5 | 1.47 | 0.2386 |
| **Region** | 1 | 835 | 23.32 | <.0001 |
| **Age*Region** | 1 | 834 | 2.88 | 0.0902 |
| **Dx*Region** | 1 | 835 | 71.34 | <.0001 |
| **Comp** | 1 | 835 | 134.04 | <.0001 |
| **Age*Comp** | 1 | 834 | 16.04 | <.0001 |
| **Dx*Comp** | 1 | 835 | 2.59 | 0.1079 |
| **Region*Comp** | 1 | 835 | 1.32 | 0.2503 |
| **Age*Dx*Region** | 1 | 834 | 36.16 | <.0001 |
| **Age*Dx*Comp** | 1 | 834 | 21.30 | <.0001 |
| **Age*Region*Comp** | 1 | 835 | 21.96 | <.0001 |

**Supplementary Table S7B.** Parameter estimates for effect of diagnosis, region, white matter component and size class on axon density from a linear mixed effect model. The 3-way interaction Region*Comp*Dx was not statistically significant (p= 0.49) and was not retained in the final model shown here.

| **Effect** | **Dx** | **Region** | **Comp** | **Estimate** | **Standard Error** | **DF** | **t Value** | **Pr > \|t\|** |
| --- | --- | --- | --- | --- | --- | --- | --- | --- |
| **Intercept** |  |  |  | 0.5452 | 0.04647 | 23.5 | 11.73 | <.0001 |
| **Age** |  |  |  | -0.00533 | 0.002015 | 23.3 | -2.64 | 0.0144 |
| **Dx** | ASD |  |  | -0.00086 | 0.06357 | 23.6 | -0.01 | 0.9893 |
| **Age*Dx** | ASD |  |  | 0.002526 | 0.002660 | 23.1 | 0.95 | 0.3522 |
| **Region** |  | FG |  | 0.02600 | 0.01846 | 834 | 1.41 | 0.1594 |
| **Age*Region** |  | FG |  | 0.000151 | 0.000779 | 833 | 0.19 | 0.8461 |
| **Dx*Region** | ASD | FG |  | -0.1783 | 0.02111 | 835 | -8.45 | <.0001 |
| **Comp** |  |  | D | -0.1513 | 0.01830 | 834 | -8.27 | <.0001 |
| **Age*Comp** |  |  | D | 0.005816 | 0.000796 | 834 | 7.31 | <.0001 |
| **Dx*Comp** | ASD |  | D | 0.03401 | 0.02113 | 835 | 1.61 | 0.1079 |
| **Region*Comp** |  | FG | D | 0.02424 | 0.02106 | 835 | 1.15 | 0.2503 |
| **Age*Dx*Region** | ASD | FG |  | 0.005265 | 0.000875 | 834 | 6.01 | <.0001 |
| **Age*Dx*Comp** | ASD |  | D | -0.00404 | 0.000876 | 834 | -4.62 | <.0001 |
| **Age*Region*Comp** |  | FG | D | -0.00408 | 0.000871 | 835 | -4.69 | <.0001 |

**Supplementary Table S8.** Slopes by diagnosis of the relationship between axon density and age for small and medium axons in the STG and FG. Comparisons of slopes between NT and ASD by size class are also provided. For STG SWM, slopes for both ASD and NT were negative and significantly different from zero. In FG, slopes differed significantly in ASD and NT groups for small and medium axons; positive slopes in ASD suggest less pruning of axons with age.

| **Region and Compartment** | **Label** | **Estimate** | **Standard Error** | **DF** | **t Value** | **Pr > \|t\|** | **Lower** | **Upper** |
| --- | --- | --- | --- | --- | --- | --- | --- | --- |
| **STG**  **SWM** | NT Medium Slope | -0.00101 | 0.000613 | 37.6 | -1.65 | 0.1065 | -0.00226 | 0.000228 |
|  | **NT Small Slope** | **-0.00403** | **0.000613** | **37.6** | **-6.57** | **<.0001***** | **-0.00527** | **-0.00279** |
|  | ASD Medium Slope | -0.00066 | 0.000582 | 37.6 | -1.13 | 0.2669 | -0.00183 | 0.000523 |
|  | **ASD Small Slope** | **-0.00319** | **0.000582** | **37.6** | **-5.48** | **<.0001***** | **-0.00437** | **-0.00201** |
|  | Small: ASD vs. NT | 0.000841 | 0.000846 | 37.6 | 0.99 | 0.3266 | -0.00087 | 0.002553 |
|  | Medium: ASD vs. NT | 0.000358 | 0.000846 | 37.6 | 0.42 | 0.6743 | -0.00135 | 0.002070 |
| **STG**  **DWM** | NT Medium Slope | 0.000167 | 0.000496 | 37.6 | 0.34 | 0.7387 | -0.00084 | 0.001170 |
|  | **NT Small Slope** | **0.001771** | **0.000496** | **37.6** | **3.57** | **0.0010**** | **0.00077** | **0.002774** |
|  | ASD Medium Slope | 0.000382 | 0.000409 | 37.6 | 0.93 | 0.3572 | -0.00045 | 0.001211 |
|  | **ASD Small Slope** | **-0.00142** | **0.000409** | **37.6** | **-3.46** | **0.0014**** | **-0.00224** | **-0.00059** |
|  | **Small: ASD vs. NT** | **-0.00319** | **0.000643** | **37.6** | **-4.96** | **<.0001***** | **-0.00449** | **-0.00188** |
|  | Medium: ASD vs. NT | 0.000215 | 0.000643 | 37.6 | 0.33 | 0.7397 | -0.00109 | 0.001517 |
| **FG**  **SWM** | **NT Medium Slope** | **-0.00170** | **0.000712** | **28.9** | **-2.38** | **0.0241*** | **-0.00315** | **-0.00024** |
|  | **NT Small Slope** | **-0.00243** | **0.000712** | **28.9** | **-3.41** | **0.0019**** | **-0.00388** | **-0.00097** |
|  | **ASD Medium Slope** | **0.002062** | **0.000622** | **28.9** | **3.31** | **0.0025**** | **0.000789** | **0.003335** |
|  | ASD Small Slope | 0.000022 | 0.000622 | 28.9 | 0.03 | 0.9724 | -0.00125 | 0.001295 |
|  | **Small: ASD vs. NT** | **0.002450** | **0.000946** | **28.9** | **2.59** | **0.0149*** | **0.000515** | **0.004384** |
|  | **Medium: ASD vs. NT** | **0.003757** | **0.000946** | **28.9** | **3.97** | **0.0004***** | **0.001823** | **0.005691** |
| **FG**  **DWM** | NT Medium Slope | -0.00028 | 0.000648 | 30.8 | -0.43 | 0.6692 | -0.00160 | 0.001043 |
|  | **NT Small Slope** | **-0.00210** | **0.000648** | **30.8** | **-3.25** | **0.0028**** | **-0.00343** | **-0.00078** |
|  | ASD Medium Slope | 0.000593 | 0.000568 | 30.8 | 1.04 | 0.3054 | -0.00057 | 0.001752 |
|  | ASD Small Slope | -0.00080 | 0.000568 | 30.8 | -1.40 | 0.1704 | -0.00196 | 0.000362 |
|  | Small: ASD vs. NT | 0.001306 | 0.000862 | 30.8 | 1.52 | 0.1399 | -0.00045 | 0.003065 |
|  | Medium: ASD vs. NT | 0.000872 | 0.000862 | 30.8 | 1.01 | 0.3196 | -0.00089 | 0.002631 |

**Supplementary Tables S9A-B.** Full results of linear mixed effect model evaluating myelin thickness versus diagnosis, region, compartment and axon size class.

**Supplementary Table S9A.** Type 3 statistics from linear mixed effect model evaluating myelin thickness versus diagnosis (Dx), region (Region), compartment (Comp), axon size class (Size). The 3-way interactions Region*Comp*Dx and Region*Dx*Size were not statistically significant (p= 0.297 and p= 0.057, respectively) and were not retained in the final model shown here.

| **Effect** | **Num DF** | **Den DF** | **F Value** | **Pr > F** |
| --- | --- | --- | --- | --- |
| Diagnosis | 1 | 25.7 | 1.97 | 0.1729 |
| CorticalRegion | 1 | 8940 | 6.82 | 0.0090 |
| CorticalRe*Diagnosis | 1 | 8947 | 6.07 | 0.0138 |
| Compartment | 1 | 8948 | 48.74 | <.0001 |
| Compartmen*Diagnosis | 1 | 8947 | 6.88 | 0.0087 |
| CorticalR*Compartmen | 1 | 8951 | 24.02 | <.0001 |
| Size | 3 | 8938 | 251.18 | <.0001 |
| Diagnosis*Size | 3 | 8938 | 3.91 | 0.0083 |
| CorticalRegion*Size | 3 | 8934 | 0.81 | 0.4887 |
| Compartment*Size | 3 | 8934 | 1.31 | 0.2708 |
| Cortica*Compart*Size | 3 | 8935 | 6.07 | 0.0004 |
| Compart*Diagnos*Size | 3 | 8934 | 4.37 | 0.0044 |

**Supplementary Table S9B.** Parameter estimates for effect of diagnosis, region, white matter component and size class on myelin thickness from a linear mixed effect model. The 3-way interactions Region*Comp*Dx and Region*Dx*Size were not statistically significant (p= 0.297 and p= 0.057, respectively) and were not retained in the final model shown here.

| **Effect** | **Region** | **Compartment** | **Diagnosis** | **Size** | **Estimate** | **Standard Error** | **DF** | **t Value** | **Pr > \|t\|** |
| --- | --- | --- | --- | --- | --- | --- | --- | --- | --- |
| **Intercept** |  |  |  |  | 0.2497 | 0.01054 | 99 | 23.69 | <.0001 |
| **Diagnosis** |  |  | ASD |  | -0.04121 | 0.01529 | 118 | -2.70 | 0.0081 |
| **CorticalRegion** | FG |  |  |  | -0.01556 | 0.01078 | 8936 | -1.44 | 0.1491 |
| **CorticalRe*Diagnosis** | FG |  | ASD |  | 0.008113 | 0.003294 | 8947 | 2.46 | 0.0138 |
| **Compartment** |  | D |  |  | -0.02070 | 0.01052 | 8941 | -1.97 | 0.0490 |
| **Compartmen*Diagnosis** |  | D | ASD |  | 0.02474 | 0.01400 | 8935 | 1.77 | 0.0771 |
| **CorticalR*Compartmen** | FG | D |  |  | 0.03565 | 0.01360 | 8936 | 2.62 | 0.0088 |
| **Size** |  |  |  | L | -0.03126 | 0.008630 | 8939 | -3.62 | 0.0003 |
| **Size** |  |  |  | M | -0.07051 | 0.008189 | 8944 | -8.61 | <.0001 |
| **Size** |  |  |  | S | -0.1063 | 0.008401 | 8949 | -12.65 | <.0001 |
| **Diagnosis*Size** |  |  | ASD | L | 0.007058 | 0.01221 | 8934 | 0.58 | 0.5631 |
| **Diagnosis*Size** |  |  | ASD | M | 0.02475 | 0.01171 | 8935 | 2.11 | 0.0345 |
| **Diagnosis*Size** |  |  | ASD | S | 0.03492 | 0.01192 | 8937 | 2.93 | 0.0034 |
| **CorticalRegion*Size** | FG |  |  | L | -0.00611 | 0.01177 | 8934 | -0.52 | 0.6040 |
| **CorticalRegion*Size** | FG |  |  | M | 0.01459 | 0.01124 | 8935 | 1.30 | 0.1942 |
| **CorticalRegion*Size** | FG |  |  | S | 0.01722 | 0.01142 | 8936 | 1.51 | 0.1317 |
| **Compartment*Size** |  | D |  | L | 0.006984 | 0.01212 | 8936 | 0.58 | 0.5646 |
| **Compartment*Size** |  | D |  | M | 0.03520 | 0.01145 | 8939 | 3.08 | 0.0021 |
| **Compartment*Size** |  | D |  | S | 0.03580 | 0.01171 | 8941 | 3.06 | 0.0022 |
| **Cortica*Compart*Size** | FG | D |  | L | 0.002008 | 0.01531 | 8934 | 0.13 | 0.8956 |
| **Cortica*Compart*Size** | FG | D |  | M | -0.02648 | 0.01448 | 8935 | -1.83 | 0.0674 |
| **Cortica*Compart*Size** | FG | D |  | S | -0.03265 | 0.01477 | 8936 | -2.21 | 0.0271 |
| **Compart*Diagnos*Size** |  | D | ASD | L | 0.000069 | 0.01567 | 8934 | 0.00 | 0.9965 |
| **Compart*Diagnos*Size** |  | D | ASD | M | -0.02255 | 0.01485 | 8934 | -1.52 | 0.1288 |
| **Compart*Diagnos*Size** |  | D | ASD | S | -0.02985 | 0.01515 | 8935 | -1.97 | 0.0488 |

**Supplementary Table S10.** Results of planned contrasts comparing mean myelin thickness between diagnoses for each size class, region and sub-region. Negative slope estimates indicate lower myelin thickness in ASD as compared to NT.

| **Label** | **Estimate** | **Standard Error** | **DF** | **t Value** | **Pr > \|t\|** |
| --- | --- | --- | --- | --- | --- |
| Large ASD vs. NT for DWM In STG | -0.00934 | 0.01156 | 38.7 | -0.81 | 0.4243 |
| Medium ASD vs. NT for DWM In STG | -0.01426 | 0.01099 | 31.6 | -1.30 | 0.2039 |
| Small ASD vs. NT for DWM In STG | -0.01140 | 0.01116 | 33.6 | -1.02 | 0.3143 |
| XL ASD vs. NT for DWM In STG | -0.01646 | 0.01333 | 68.1 | -1.24 | 0.2209 |
| **Large ASD vs. NT for SWM In STG** | **-0.03415** | **0.01148** | **37.6** | **-2.97** | **0.0051**** |
| Medium ASD vs. NT for SWM In STG | -0.01645 | 0.01090 | 30.5 | -1.51 | 0.1415 |
| Small ASD vs. NT for SWM In STG | -0.00629 | 0.01107 | 32.4 | -0.57 | 0.5737 |
| **XL ASD vs. NT for SWM In STG** | **-0.04121** | **0.01529** | **118** | **-2.70** | **0.0081**** |
| Large ASD vs. NT for DWM In FG | -0.00122 | 0.01157 | 38.8 | -0.11 | 0.9163 |
| Medium ASD vs. NT for DWM In FG | -0.00615 | 0.01101 | 31.8 | -0.56 | 0.5805 |
| Small ASD vs. NT for DWM In FG | -0.00329 | 0.01126 | 34.7 | -0.29 | 0.7718 |
| XL ASD vs. NT for DWM In FG | -0.00835 | 0.01330 | 67.6 | -0.63 | 0.5322 |
| **Large ASD vs. NT for SWM In FG** | **-0.02604** | **0.01140** | **36.5** | **-2.28** | **0.0283*** |
| Medium ASD vs. NT for SWM In FG | -0.00834 | 0.01085 | 30 | -0.77 | 0.4482 |
| Small ASD vs. NT for SWM In FG | 0.001822 | 0.01110 | 32.8 | 0.16 | 0.8706 |
| **XL ASD vs. NT for SWM In FG** | **-0.03309** | **0.01524** | **116** | **-2.17** | **0.0319*** |

**Supplementary Table S11.** G-ratio means and standard deviations between diagnoses across axon classes.

|  | **Overall Mean (SD)** | **NT Mean (SD)** | **ASD Mean (SD)** |
| --- | --- | --- | --- |
| Small (<0.35 µM) | .494 (±.098) | .490 (±.095) | .497 (±.100) |
| Medium (0.35-.7 µM) | .601 (±.101) | .596 (±.097) | .606 (±.104) |
| Large (.7-1.4 µM) | .726 (±.097) | .727 (±.095) | .724 (±.099) |
| Extra Large (+1.4 µM) | .812 (±.067) | .807 (±.072) | .818 (±.095) |

**Supplementary Table S12A-B.** Full results of linear mixed effect model evaluating myelin thickness versus age, diagnosis, region, and compartment.

**Supplementary Table S12A.** Type 3 statistics from linear mixed effect model evaluating myelin thickness versus age, diagnosis (Dx), region (Region), and compartment (Comp). The 3-way interactions age*region*subregion and diagnosis*region*subregion were not statistically significant (p = 0.814 and p = 0.500, respectively) and were not retained in the final model shown here.

| **Effect** | **Num DF** | **Den DF** | **F Value** | **Pr > F** |
| --- | --- | --- | --- | --- |
| Age | 1 | 22.1 | 1.13 | 0.2992 |
| Diagnosis | 1 | 22.2 | 0.27 | 0.6106 |
| Age*Diagnosis | 1 | 22.1 | 2.06 | 0.1648 |
| CorticalRegion | 1 | 8952 | 4.75 | 0.0294 |
| Age*CorticalRegion | 1 | 8953 | 0.17 | 0.6822 |
| Diagnosis*CorticalRe | 1 | 8951 | 0.87 | 0.3511 |
| Compartment | 1 | 8958 | 2.78 | 0.0953 |
| Age*Compartment | 1 | 8953 | 19.51 | <.0001 |
| Diagnosis*Compartmen | 1 | 8958 | 7.75 | 0.0054 |
| CorticalR*Compartmen | 1 | 8967 | 22.23 | <.0001 |
| Age*Diagnos*Compartm | 1 | 8954 | 18.66 | <.0001 |
| Age*Diagnos*Cortical | 1 | 8952 | 5.41 | 0.0201 |

**Supplementary Table S12B.** Parameter estimates for the final linear mixed effect model relating myelin thickness to age by diagnosis, region and subregion.

| **Effect** | **Diagnosis** | **CorticalRegion** | **Compartment** | **Estimate** | **Standard Error** | **DF** | **t Value** | **Pr > \|t\|** |
| --- | --- | --- | --- | --- | --- | --- | --- | --- |
| **Intercept** |  |  |  | 0.1563 | 0.01626 | 24.2 | 9.61 | <.0001 |
| **Age** |  |  |  | 0.001261 | 0.000703 | 23.8 | 1.79 | 0.0858 |
| **Diagnosis** | ASD |  |  | 0.02393 | 0.02229 | 24.6 | 1.07 | 0.2934 |
| **Age*Diagnosis** | ASD |  |  | -0.00223 | 0.000927 | 24 | -2.40 | 0.0245 |
| **CorticalRegion** |  | FG |  | 0.002271 | 0.005425 | 8958 | 0.42 | 0.6755 |
| **Age*CorticalRegion** |  | FG |  | -0.00026 | 0.000218 | 8955 | -1.22 | 0.2239 |
| **Diagnosis*CorticalRe** | ASD | FG |  | -0.00633 | 0.006789 | 8951 | -0.93 | 0.3511 |
| **Compartment** |  |  | D | 0.006986 | 0.005421 | 8955 | 1.29 | 0.1976 |
| **Age*Compartment** |  |  | D | 0.000013 | 0.000216 | 8952 | 0.06 | 0.9506 |
| **Diagnosis*Compartmen** | ASD |  | D | -0.01909 | 0.006860 | 8958 | -2.78 | 0.0054 |
| **CorticalR*Compartmen** |  | FG | D | 0.01656 | 0.003513 | 8967 | 4.71 | <.0001 |
| **Age*Diagnos*Compartm** | ASD |  | D | 0.001202 | 0.000278 | 8954 | 4.32 | <.0001 |
| **Age*Diagnos*Cortical** | ASD | FG |  | 0.000642 | 0.000276 | 8952 | 2.33 | 0.0201 |

**Supplementary Table S13.** Results of planned contrasts comparing slopes of myelin thickness vs. age between ASD and NT subjects within each region and subregion.

| **Label** | **Estimate** | **SE** | **DF** | **t Value** | **Pr > \|t\|** | **Lower** | **Upper** |
| --- | --- | --- | --- | --- | --- | --- | --- |
| NT STG SWM | 0.001261 | 0.000703 | 23.8 | 1.79 | 0.0858 | -0.00019 | 0.002713 |
| NT STG DWM | 0.001274 | 0.000708 | 24.5 | 1.80 | 0.0843 | -0.00019 | 0.002734 |
| NT FG SWM | 0.000996 | 0.000709 | 24.6 | 1.40 | 0.1726 | -0.00047 | 0.002457 |
| NT FG DWM | 0.001009 | 0.000708 | 24.4 | 1.43 | 0.1664 | -0.00045 | 0.002468 |
| ASD STG SWM | -0.00097 | 0.000604 | 24.3 | -1.60 | 0.1229 | -0.00221 | 0.000281 |
| ASD STG DWM | 0.000249 | 0.000602 | 23.9 | 0.41 | 0.6828 | -0.00099 | 0.001492 |
| ASD FG SWM | -0.00059 | 0.000597 | 23.1 | -0.99 | 0.3348 | -0.00182 | 0.000647 |
| ASD FG DWM | 0.000627 | 0.000606 | 24.6 | 1.03 | 0.3113 | -0.00062 | 0.001877 |
| **STG SWM: ASD vs. NT** | **-0.00223** | **0.000927** | **24** | **-2.40** | **0.0245*** | **-0.00414** | **-0.00031** |
| STG DWM: ASD vs. NT | -0.00102 | 0.000929 | 24.2 | -1.10 | 0.2810 | -0.00294 | 0.000893 |
| FG SWM: ASD vs. NT | -0.00158 | 0.000927 | 24 | -1.71 | 0.1004 | -0.00350 | 0.000329 |
| FG DWM: ASD vs. NT | -0.00038 | 0.000932 | 24.5 | -0.41 | 0.6851 | -0.00230 | 0.001539 |

**Supplementary Tables S14A/B.** Full results of linear mixed effect model evaluating myelin thickness versus diagnosis, region, compartment and axon size class.

**Supplementary Table S14A.** Type 3 statistics from linear mixed effect model evaluating myelin thickness versus diagnosis (Dx), region (Region), compartment (Comp), axon size class (Size). The 3-way interactions Region*Comp*Dx and Region*Dx*Size were not statistically significant (p= 0.297 and p= 0.057, respectively) and were not retained in the final model shown here.

| **Effect** | **Num DF** | **Den DF** | **F Value** | **Pr > F** |
| --- | --- | --- | --- | --- |
| Diagnosis | 1 | 25.7 | 1.97 | 0.1729 |
| CorticalRegion | 1 | 8940 | 6.82 | 0.0090 |
| CorticalRe*Diagnosis | 1 | 8947 | 6.07 | 0.0138 |
| Compartment | 1 | 8948 | 48.74 | <.0001 |
| Compartmen*Diagnosis | 1 | 8947 | 6.88 | 0.0087 |
| CorticalR*Compartmen | 1 | 8951 | 24.02 | <.0001 |
| Size | 3 | 8938 | 251.18 | <.0001 |
| Diagnosis*Size | 3 | 8938 | 3.91 | 0.0083 |
| CorticalRegion*Size | 3 | 8934 | 0.81 | 0.4887 |
| Compartment*Size | 3 | 8934 | 1.31 | 0.2708 |
| Cortica*Compart*Size | 3 | 8935 | 6.07 | 0.0004 |
| Compart*Diagnos*Size | 3 | 8934 | 4.37 | 0.0044 |

**Supplementary Table 14B.** Parameter estimates for effect of diagnosis, region, white matter component and size class on myelin thickness from a linear mixed effect model.

| **Effect** | **CorticalRegion** | **Compartment** | **Diagnosis** | **Size** | **Estimate** | **Standard Error** | **DF** | **t Value** | **Pr > \|t\|** |
| --- | --- | --- | --- | --- | --- | --- | --- | --- | --- |
| **Intercept** |  |  |  |  | 0.2497 | 0.01054 | 99 | 23.69 | <.0001 |
| **Diagnosis** |  |  | ASD |  | -0.04121 | 0.01529 | 118 | -2.70 | 0.0081 |
| **CorticalRegion** | FG |  |  |  | -0.01556 | 0.01078 | 8936 | -1.44 | 0.1491 |
| **CorticalRe*Diagnosis** | FG |  | ASD |  | 0.008113 | 0.003294 | 8947 | 2.46 | 0.0138 |
| **Compartment** |  | D |  |  | -0.02070 | 0.01052 | 8941 | -1.97 | 0.0490 |
| **Compartmen*Diagnosis** |  | D | ASD |  | 0.02474 | 0.01400 | 8935 | 1.77 | 0.0771 |
| **CorticalR*Compartmen** | FG | D |  |  | 0.03565 | 0.01360 | 8936 | 2.62 | 0.0088 |
| **Size** |  |  |  | L | -0.03126 | 0.008630 | 8939 | -3.62 | 0.0003 |
| **Size** |  |  |  | M | -0.07051 | 0.008189 | 8944 | -8.61 | <.0001 |
| **Size** |  |  |  | S | -0.1063 | 0.008401 | 8949 | -12.65 | <.0001 |
| **Diagnosis*Size** |  |  | ASD | L | 0.007058 | 0.01221 | 8934 | 0.58 | 0.5631 |
| **Diagnosis*Size** |  |  | ASD | M | 0.02475 | 0.01171 | 8935 | 2.11 | 0.0345 |
| **Diagnosis*Size** |  |  | ASD | S | 0.03492 | 0.01192 | 8937 | 2.93 | 0.0034 |
| **CorticalRegion*Size** | FG |  |  | L | -0.00611 | 0.01177 | 8934 | -0.52 | 0.6040 |
| **CorticalRegion*Size** | FG |  |  | M | 0.01459 | 0.01124 | 8935 | 1.30 | 0.1942 |
| **CorticalRegion*Size** | FG |  |  | S | 0.01722 | 0.01142 | 8936 | 1.51 | 0.1317 |
| **Compartment*Size** |  | D |  | L | 0.006984 | 0.01212 | 8936 | 0.58 | 0.5646 |
| **Compartment*Size** |  | D |  | M | 0.03520 | 0.01145 | 8939 | 3.08 | 0.0021 |
| **Compartment*Size** |  | D |  | S | 0.03580 | 0.01171 | 8941 | 3.06 | 0.0022 |
| **Cortica*Compart*Size** | FG | D |  | L | 0.002008 | 0.01531 | 8934 | 0.13 | 0.8956 |
| **Cortica*Compart*Size** | FG | D |  | M | -0.02648 | 0.01448 | 8935 | -1.83 | 0.0674 |
| **Cortica*Compart*Size** | FG | D |  | S | -0.03265 | 0.01477 | 8936 | -2.21 | 0.0271 |
| **Compart*Diagnos*Size** |  | D | ASD | L | 0.000069 | 0.01567 | 8934 | 0.00 | 0.9965 |
| **Compart*Diagnos*Size** |  | D | ASD | M | -0.02255 | 0.01485 | 8934 | -1.52 | 0.1288 |
| **Compart*Diagnos*Size** |  | D | ASD | S | -0.02985 | 0.01515 | 8935 | -1.97 | 0.0488 |

**Supplementary Table S15.** Slopes by diagnosis of the relationship between myelin thickness and age for each region and subregion in large and XL axons in temporal white matter. Estimates based on linear mixed effect model relating axon density to age, axon size and diagnosis. Comparisons of slopes between NT and ASD are also provided.

| **Region of Interest** | **Label** | **Estimate** | **Standard Error** | **DF** | **t Value** | **Pr > \|t\|** | **Lower** | **Upper** |
| --- | --- | --- | --- | --- | --- | --- | --- | --- |
| **STG**  **SWM** | **NT Large** | **0.003260** | **0.000931** | **24.5** | **3.50** | **0.0018**** | **0.001341** | **0.005179** |
|  | **NT X Large** | **0.002706** | **0.001273** | **83.9** | **2.13** | **0.0365*** | **0.000174** | **0.005238** |
|  | ASD Large | -0.00168 | 0.000920 | 29 | -1.83 | 0.0777 | -0.00356 | 0.000199 |
|  | ASD X Large | -0.00064 | 0.001557 | 226 | -0.41 | 0.6807 | -0.00371 | 0.002427 |
|  | **Large: ASD vs. NT** | **-0.00494** | **0.001308** | **26.6** | **-3.78** | **0.0008***** | **-0.00763** | **-0.00226** |
|  | X Large: ASD vs. NT | -0.00335 | 0.002012 | 144 | -1.66 | 0.0983 | -0.00732 | 0.000629 |
| **STG**  **DWM** | NT Large | 0.001360 | 0.000868 | 28.2 | 1.57 | 0.1284 | -0.00042 | 0.003137 |
|  | **NT X Large** | **0.003218** | **0.001070** | **64.8** | **3.01** | **0.0037**** | **0.001081** | **0.005355** |
|  | ASD Large | 0.000807 | 0.000749 | 28.6 | 1.08 | 0.2899 | -0.00072 | 0.002339 |
|  | **ASD X Large** | **0.001983** | **0.000975** | **81.3** | **2.03** | **0.0452*** | **0.000043** | **0.003923** |
|  | Large: ASD vs. NT | -0.00055 | 0.001146 | 28.4 | -0.48 | 0.6336 | -0.00290 | 0.001794 |
|  | X Large: ASD vs. NT | -0.00123 | 0.001447 | 71.6 | -0.85 | 0.3965 | -0.00412 | 0.001651 |
| **FG**  **SWM** | NT Large | 0.000745 | 0.000816 | 28 | 0.91 | 0.3693 | -0.00093 | 0.002416 |
|  | NT X Large | 0.001992 | 0.001142 | 106 | 1.74 | 0.0839 | -0.00027 | 0.004257 |
|  | ASD Large | -0.00088 | 0.000675 | 24.9 | -1.31 | 0.2026 | -0.00227 | 0.000507 |
|  | ASD X Large | 0.000447 | 0.000901 | 78.4 | 0.50 | 0.6211 | -0.00135 | 0.002242 |
|  | Large: ASD vs. NT | -0.00163 | 0.001059 | 26.7 | -1.54 | 0.1360 | -0.00380 | 0.000546 |
|  | X Large: ASD vs. NT | -0.00155 | 0.001455 | 93.8 | -1.06 | 0.2910 | -0.00443 | 0.001344 |
| **FG**  **DWM** | NT Large | 0.001238 | 0.001430 | 24.1 | 0.87 | 0.3951 | -0.00171 | 0.004188 |
|  | NT X Large | 0.003072 | 0.001597 | 37.5 | 1.92 | 0.0621 | -0.00016 | 0.006306 |
|  | ASD Large | -0.00046 | 0.001264 | 25.4 | -0.37 | 0.7161 | -0.00307 | 0.002136 |
|  | **ASD X Large** | **0.003753** | **0.001424** | **40.8** | **2.64** | **0.0118*** | **0.000877** | **0.006628** |
|  | Large: ASD vs. NT | -0.00170 | 0.001908 | 24.7 | -0.89 | 0.3809 | -0.00564 | 0.002230 |
|  | X Large: ASD vs. NT | 0.000681 | 0.002140 | 38.9 | 0.32 | 0.7519 | -0.00365 | 0.005009 |
