# Supplementary material for: Age-related differences in axon pruning and myelination may alter neural signaling in autism spectrum disorder
Source: Mol Autism. 2025 Oct 23;16:53. doi: 10.1186/s13229-025-00684-y (PMC12548170; doi:10.1186/s13229-025-00684-y)

Supplementary Figure S1. Axon density vs. age (years) in superior temporal gyrus (STG) in superficial (SWM) and deep white matter (DWM) by axon size class. Slopes and p-values for small and medium axons are available in Supplementary Table S8.


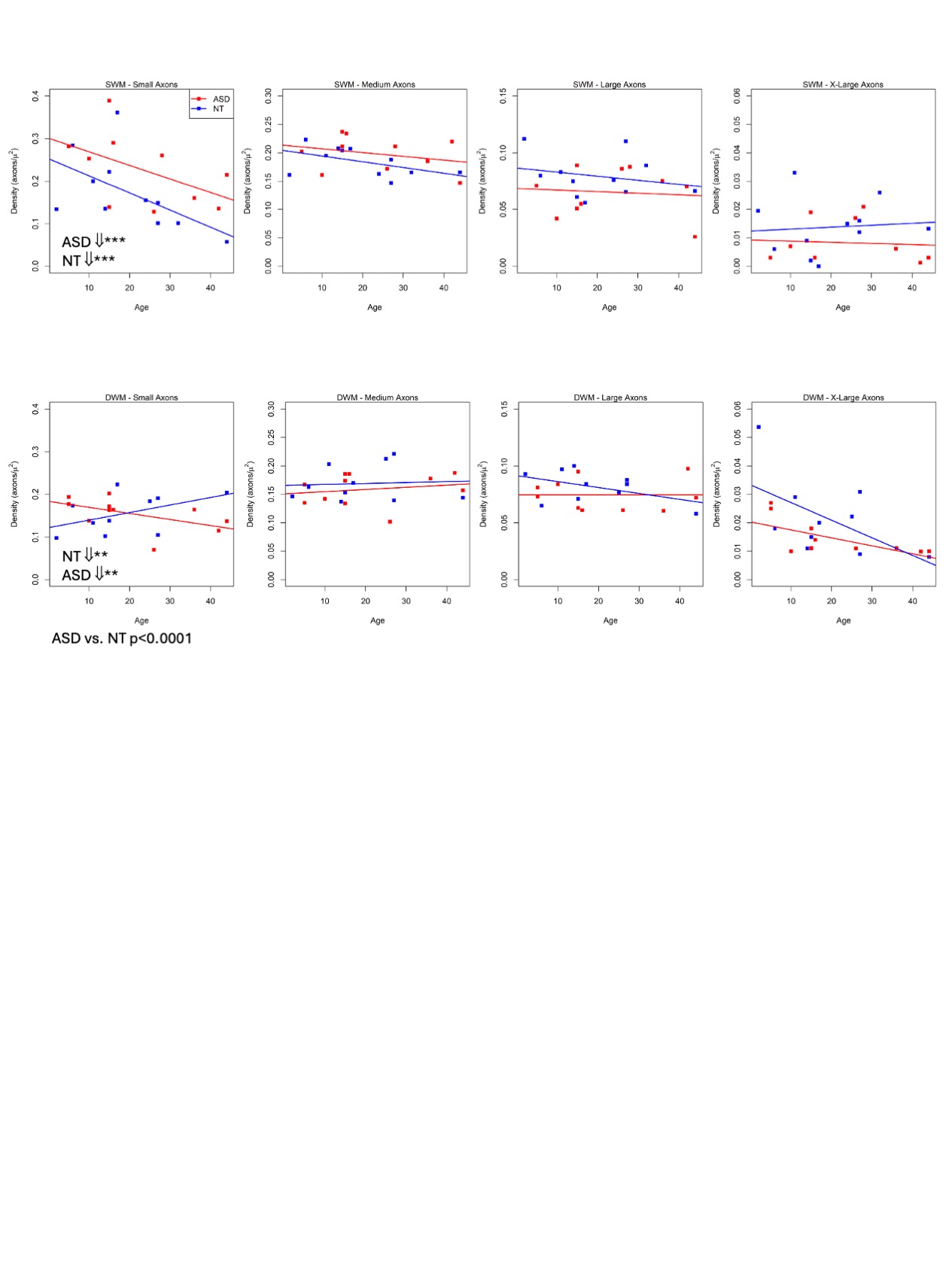


Supplementary Figure S2. Axon density vs. age (years) in fusiform gyrus (FG) in superficial (SWM) and deep white matter (DWM) by axon size class (S, M, L, XL). Slopes and p-values for small and medium axons are available in Supplementary Table S8.


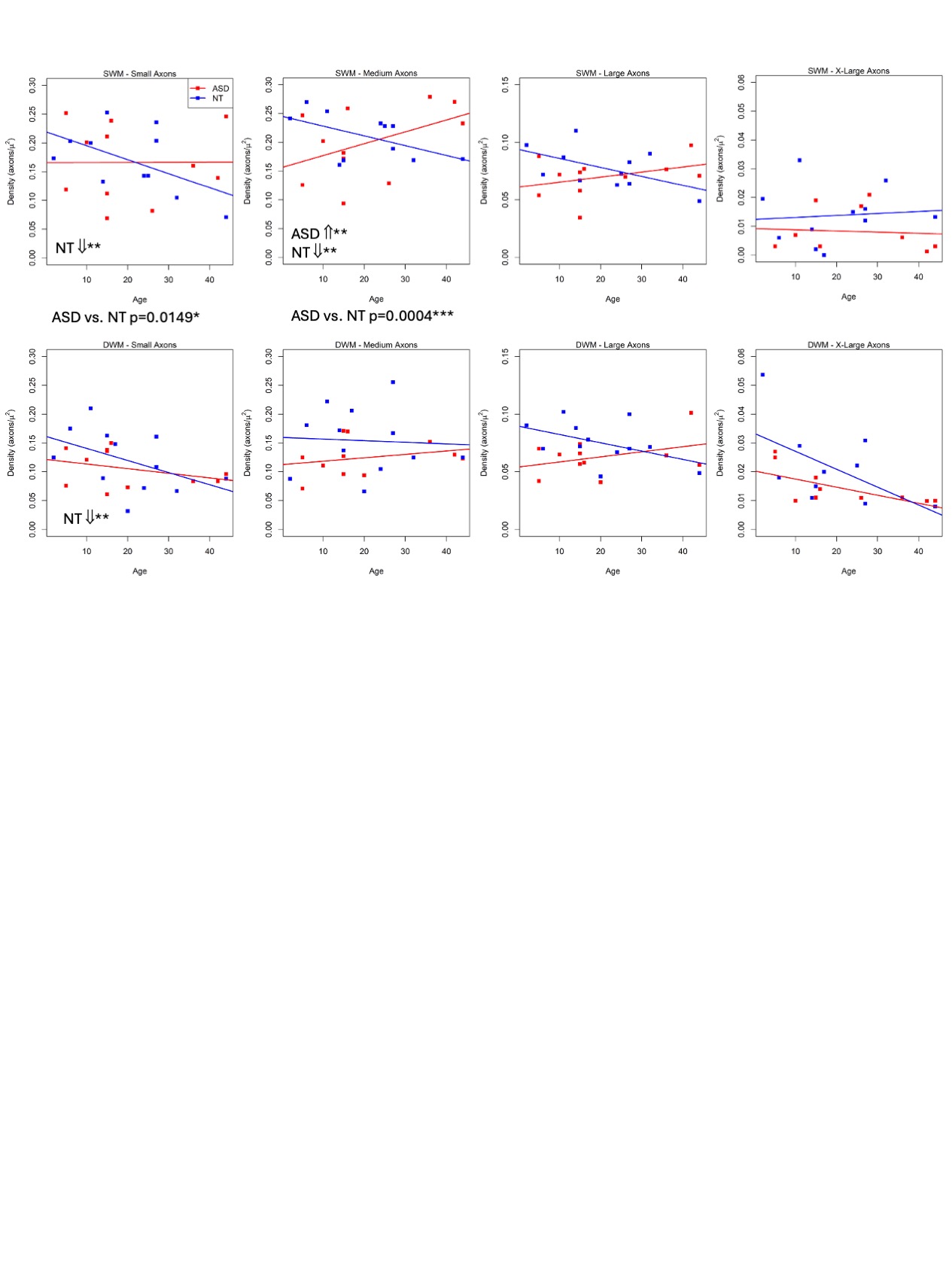


Supplementary Figure S3. Myelin thickness by age (years) in L and XL axons for SWM and DWM in STG and FG. Slopes and p-values are available in Supplementary Table S15.


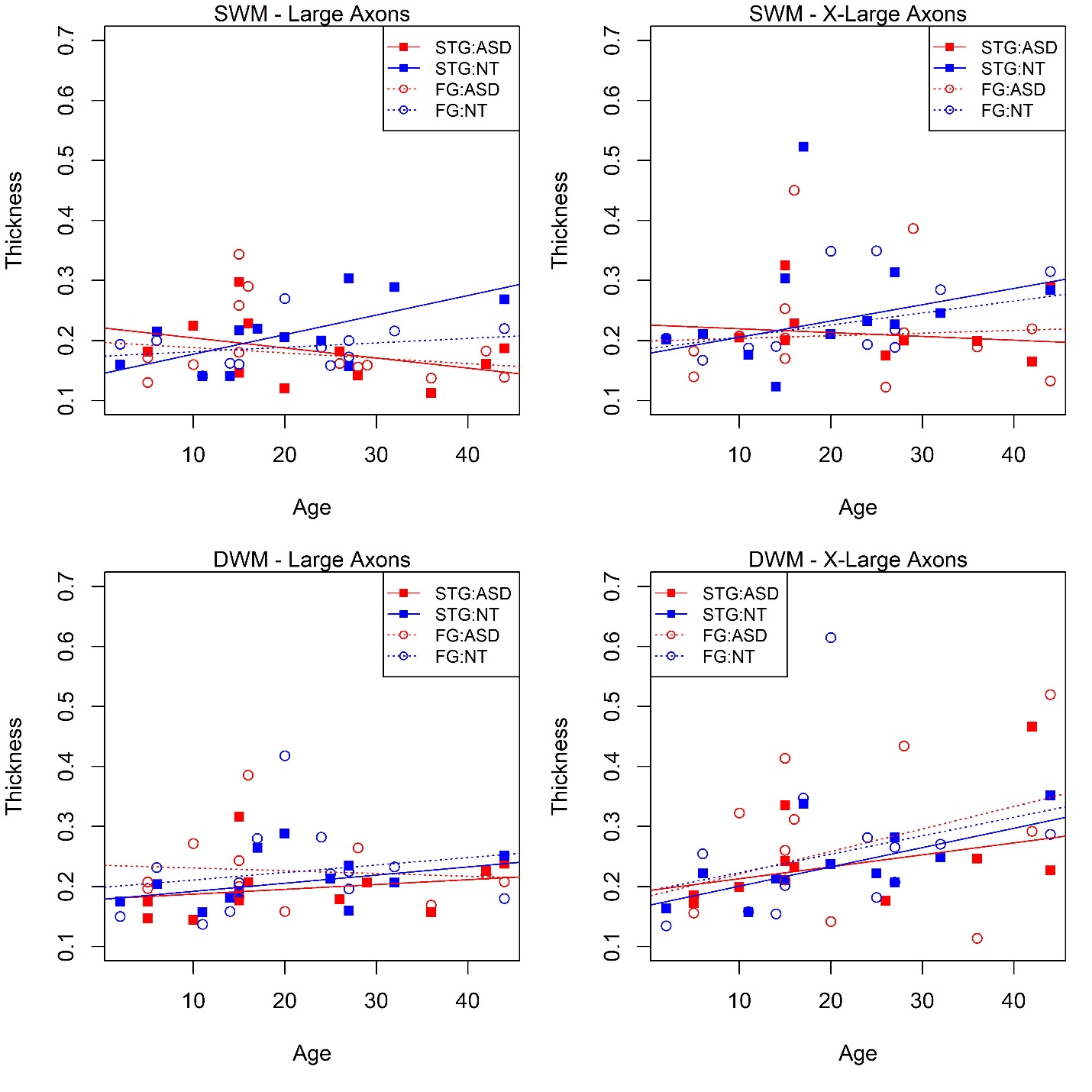


Supplementary Figure S4. G-Ratio and cumulative myelin thickness measures in STG and FG. A) Average g-Ratio measurements progressively increased from small (0-.35 uM) to extra large ((>1.4 uM) axons for both NT and ASD cases in superficial and deep white matter of STG and FG. No difference in g-Ratio was observed between NT and ASD subjects in any region or compartment. B) Cumulative plots of size within large superficial axon (.7-1.4 uM) and extra-large superficial axon (>1.4 uM) categories in STG and FG. No difference in axon size distribution was observed in FG for either size category, however, both large and extra-large axons in STG were shifted towards a smaller distribution.


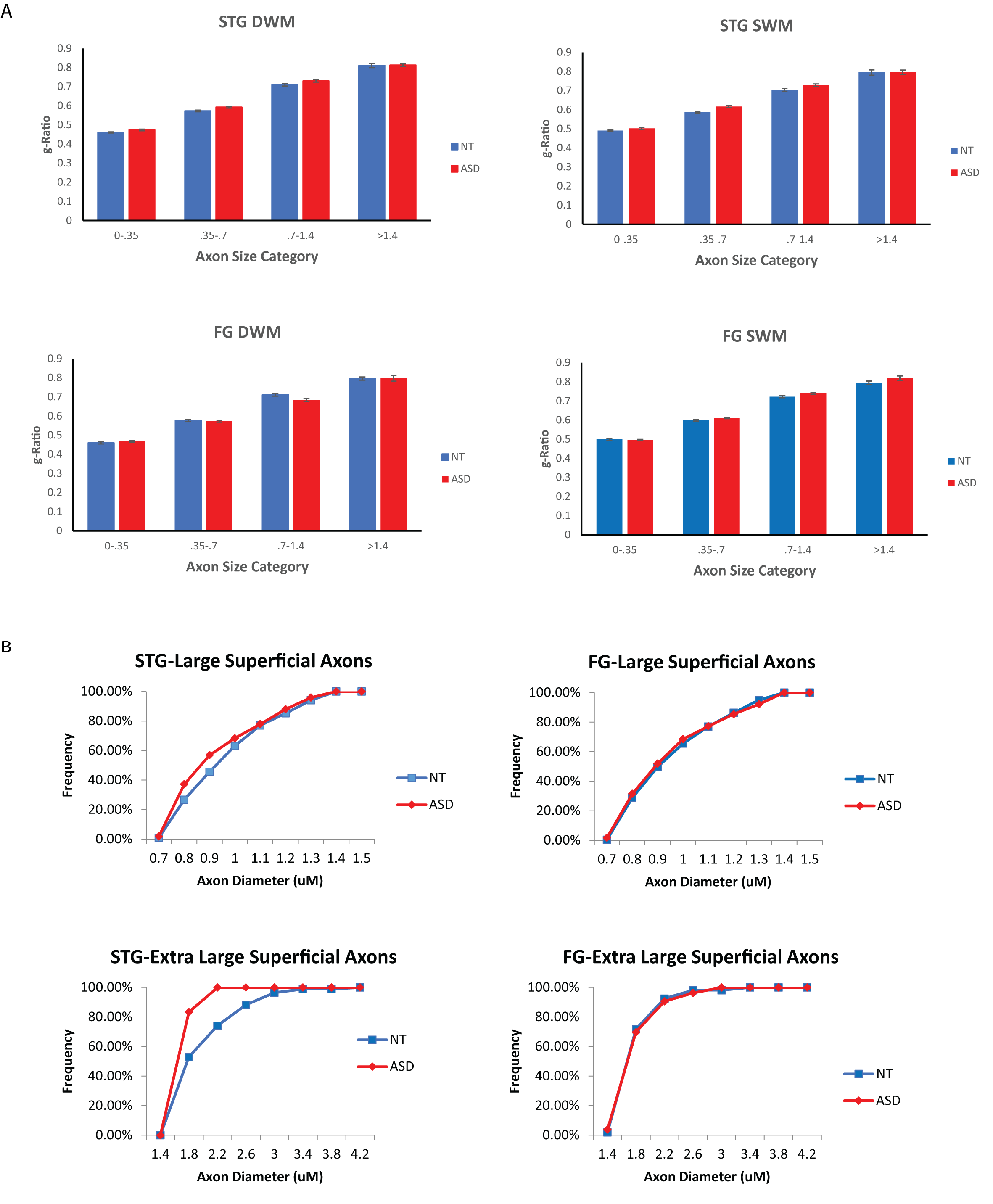

Supplement: Supplementary file 2 — Supplementary Material 2 [file 13229_2025_684_MOESM2_ESM.docx]
